# CmbZIP11 regulates *CmPMT1*/*15* affecting homogalacturonan methyl-esterification and fruit softening in melon

**DOI:** 10.1093/hr/uhaf253

**Published:** 2025-09-12

**Authors:** Haobin Pan, Yinhan Sun, Ruirui Wei, Hongyan Qi

**Affiliations:** College of Horticulture, Shenyang Agricultural University, Shenyang, Liaoning 110866, China; Key Laboratory of Protected Horticulture of Education Ministry and Liaoning Province, Shenyang, Liaoning 110866, China; Northern National & Local Joint Engineering Research Center of Horticultural Facilities Design and Application Technology (Liaoning), Shenyang, Liaoning 110866, China; College of Horticulture, Shenyang Agricultural University, Shenyang, Liaoning 110866, China; Key Laboratory of Protected Horticulture of Education Ministry and Liaoning Province, Shenyang, Liaoning 110866, China; Northern National & Local Joint Engineering Research Center of Horticultural Facilities Design and Application Technology (Liaoning), Shenyang, Liaoning 110866, China; College of Horticulture, Shenyang Agricultural University, Shenyang, Liaoning 110866, China; Key Laboratory of Protected Horticulture of Education Ministry and Liaoning Province, Shenyang, Liaoning 110866, China; Northern National & Local Joint Engineering Research Center of Horticultural Facilities Design and Application Technology (Liaoning), Shenyang, Liaoning 110866, China; College of Horticulture, Shenyang Agricultural University, Shenyang, Liaoning 110866, China; Key Laboratory of Protected Horticulture of Education Ministry and Liaoning Province, Shenyang, Liaoning 110866, China; Northern National & Local Joint Engineering Research Center of Horticultural Facilities Design and Application Technology (Liaoning), Shenyang, Liaoning 110866, China

## Abstract

Homogalacturonan (HG) methyl-esterified modification pathway genome-wide expression analysis in two texture types of oriental melon fruits ‘HDB’ (crisp) and ‘HPM’ (mealy) at different developmental stages revealed that the Golgi S-adenosyl-L-methionine transporter gene *CmGoSAMT1* and the pectin methyltransferase genes *CmPMT1* and *CmPMT15* were critical functional genes for HG methyl-esterification in melon. However, overexpression of *CmGoSAMT1* cannot significantly alter fruit hardness, whereas overexpression of *CmPMT1* and *CmPMT15* appears to cause the fruit to synthesize more highly methyl-esterified HG, which are separated from each other due to the ‘steric effect’ of methyl groups on the *C*-6 carboxyl of D-GalA, and cannot be cross-linked by Ca^2+^ to form an ‘egg-box’ matrix making it more accessible to de-methylating and degrading enzymes (CmPMEs, CmPGs, and CmPLs), thus fruit susceptible to softening. In addition, a transcription factor CmbZIP11 co-expressed with *CmPMT1* and *CmPMT15* was verified, which could activate the expression of *CmPMT1* and *CmPMT15* to regulate the methyl-esterification of HG, thereby affecting fruit softening.

## Introduction

Melon (*Cucumis melo* L.) is one of the most popular horticultural crops worldwide. It can be divided into 2 subspecies and 16 variants with a rich variation in fruit texture due to its diversity [1–3]. Melon is a climacteric fruit, which undergoes rapid softening during fruit ripening and postharvest, thus affecting its commercial value relating to transportation, storage, and shelf life. Therefore, it is important to investigate the molecular regulatory mechanism of fruit texture quality and softening for melon breeding.

Pectin is the most structurally variable and dynamic polysaccharide in the cell wall, which influences wall porosity, cell adhesion, mechanical properties, ions binding, etc. [4, 5]. Studies have shown that pectin metabolism plays a key role in fruit softening [6]. Homogalacturonan (HG) is the most abundant pectic polysaccharide (~65%) in higher plants, which is a liner homopolymer constructed from D-galacturonic acid (D-GalA) linked by α-1,4-glycosidic bond, with up to 80% of its *C*-6 position methyl-esterified and a lesser extent acetylated at *O*-2 and *O*-3 positions [7]. These modifications are critical for the biochemical properties of HG. After secretion to the extracelullar space HG may undergo de-methylation by pectin methylesterases (PMEs) and the de-methylated HG generally has two fates: (i) cross-linked by divalent cations (mainly Ca^2+^) to form a stable ‘egg-box’ matrix if at least seven consecutive carboxyl of GalA residues are de-methylated [8–10]; and (ii) hydrolyzed by pectin-degrading enzymes such as polygalacturonases (PGs) and pectate lyases (PLs) [11, 12]. Therefore, the degree of methyl-esterification (DM) of HG in middle lamella (ML) is responsible to the form of the ‘egg-box’ gel structure and intercellular adhesion is thought to be closely related to fruit softening.

Methyl-esterified modification of HG occurs in the Golgi apparatus after it is synthesized by Golgi-localized galacturonosyltransferases (GAUTs) [7, 13]. Pectin methyltransferases (PMTs) play the function to transfer the methyl from S-adenosyl-L-methionine (SAM) to the *C*-6 carboxyl of the D-GalA residue of the HG acceptor. In the process, the SAM donors in the Golgi apparatus are provided by Golgi SAM transporters (GoSAMTs), which act as a bidirectional transporter that importing SAM into the Golgi membrane while exporting the de-methylated product S-adenosyl homocysteine (SAH) [14–16]. Therefore, PMTs and GoSAMTs are the key enzymes in the methyl assembly for HG. However, current studies on PMTs and GoSAMTs are limited to *Arabidopsis thaliana* and there are no reports on their role in fruit texture.

On the other hand, the process of HG de-methyl-esterification in the cell wall is relative complex, which is synergistically regulated by PMEs, pectin methylesterase inhibitors (PMEIs), and subtilisin-like serine proteases (SBTs) [17, 18]. Among them, PMEs perform the de-methyl-esterification function, and PMEIs act as active inhibitors for PMEs, while SBTs play a role in shearing and release of the functional domain of the PMEs by recognizing specific motif between the PME domain and the PRO region (PMEI domain) [19–22]. However, the equilibrium mechanism between them is still unclear.

Most of the current studies on the HG metabolism have mainly focused on enzymes related to de-methyl-esterification and degradation pathways, However, there are no reports on the effect of HG methyl assembly by GoSAMTs and PMTs on fruit texture. Furthermore, previous study by Pan *et al*. [23] in two texture types of oriental melons ‘HDB’ (crisp) and ‘HPM’ (mealy) have demonstrated that the higher abundance of de-methyl-esterified HG in the ML and intercellular junction zones of ‘HDB’ sarcocarp tissues enable it to form more HG-Ca^2+^ cross-linked ‘egg-box’ gel structures, which leads to strong intercellular adhesion, resulting in cell rupture and release of cellular endocytic contents when tissue fracture, therefore showed a crispy and juicy fruit texture. In contrast, the lower abundance of de-methyl-esterified HG in the ML and intercellular junction zones of ‘HPM’ results in fewer HG-Ca^2+^ cross-linked ‘egg-box’ gel structures abundance, which makes it more susceptible to degradation during ripening thus leading to loss of intercellular adhesion of the sarcocarp tissue with a cell separation fracture pattern and showed a mealy, juiceless fruit texture.

Therefore, ‘HDB’ and ‘HPM’, which have significant difference in the DM of HG were used as materials in this study. By constructing comparative transcriptomes of fruits of the two melon cultivars, based on weighted gene co-expression network analysis (WGCNA) correlated with the DM of HG during fruit development, aiming to screening out the key functional genes and transcription factors (TFs) contributes to HG methyl-esterification of melon fruit. Ultimately, it is intended to elucidate the molecular regulatory mechanism of HG methyl-esterification on affecting melon fruit softening.

## Results

### C*mGoSAMT1*, *CmPMT1*, and *CmPMT15* positively correlated with methyl-esterification of HG

In order to identify the key functional genes and TFs responsible for the methyl-esterification of HG in melon fruit, the gene family members of *CmGoSAMTs* (3), *CmPMTs* (18), *CmPMEs* (49), *CmPMEIs* (69), and *CmSBTs* (41) involved in the HG methyl-esterification pathway were identified genome-wide (Table S1), and the expression of above families along with all the TFs in the HDB_vs_HPM fruit transcriptome at fruitlet, expanding, and mature stages was taken as the dataset for the WGCNA. Ultimately, the gene expression data was hierarchically clustered into seven modules. Correlation analysis between modules with hardness and DM of HG showed that the blue and turquoise modules highly positively correlated with hardness, with correlation coefficients of 0.7 and 0.67, respectively, while the red module had the highest negative correlation with hardness (−0.89). At the same time, the brown module had the highest positive correlation with the DM of HG (0.69). The green and yellow modules exhibited lower negatively correlation coefficients with the DM of HG, with −0.78 and −0.8, respectively (Fig. S2A and B).

**Figure 1:**
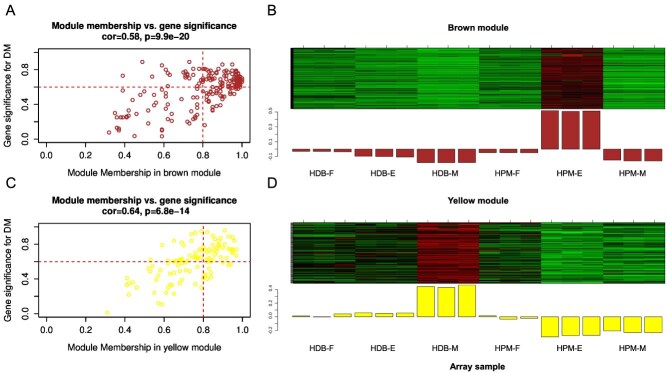
GS-MM scatter plot and expression pattern of HG methyl-esterification-related module genes in transcriptome. (A) and (C) GS-MM scatter plots of module genes significance for degree of methyl-esterification (DM) of HG and module membership. (B) and (D) Expression patterns of module genes in transcriptome of ‘HDB’ and ‘HPM’ fruits at fruitlet, expanding, and mature stages.

Subsequently, key genes with higher correlation with the DM of HG were screened out from the brown, green, and yellow modules by gene significance versus module membership (GS-MM) with the inter-module thresholds set at 0.6 and 0.8, respectively. The GS-MM scatter plots and the expression patterns of the genes in the brown module (204 genes) and yellow module (109 genes) are shown in Fig. 1. One Golgi SAM transporters gene, *CmGoSAMT1* (MELO3C005898.1), and four PMT genes, *CmPMT1* (MELO3C008535.1), *CmPMT3* (MELO3C005813.1), *CmPMT15* (MELO3C024603.1), and *CmPMT6* (MELO3C022753.1) were screened out in the brown module. One PMEI gene, *CmPMEI45* (MELO3C006266.1), and one SBT gene, *CmSBT1.7b* (MELO3C010817.1), was screened out in the yellow module. No functional genes were screened out in the green module. Ranking based on the correlation coefficients showed that *CmGoSAMT1* had the highest correlation coefficient with the DM of HG (0.865), followed by *CmPMT1*, *CmPMT3*, *CmPMT15*, and *CmPMT6* with correlation coefficients of 0.752, 0.680, 0.666, and 0.609, respectively (Table 1). Therefore, it was concluded that the *GoSAMTs* and *CmPMTs* play important roles in the methyl-esterification of HG in melon fruit. The conserved functional domains distribution of *CmGoSAMTs* and *CmPMTs* members are shown in the Figs S3 and S4, respectively. The phylogenetic relationship of GoSAMTs and PMTs between *C. melo* and *A. thaliana* are shown in the Figs S5 and S6, respectively.

**Table 1:** Correlation analysis between expression of HG methyl-esterification pathway genes and degree of methyl-esterification of HG

ID	Name	Description	Correlation	*P*-value
MELO3C005898.1	*CmGoSAMT1*	Golgi SAM transporter	0.865	0.000
MELO3C008535.1	*CmPMT1*	Pectin methyltransferase	0.752	0.000
MELO3C005813.1	*CmPMT3*	Pectin methyltransferase	0.680	0.002
MELO3C024603.1	*CmPMT15*	Pectin methyltransferase	0.666	0.003
MELO3C022753.1	*CmPMT6*	Pectin methyltransferase	0.609	0.007
MELO3C006266.1	*CmPMEI45*	Pectin methylesterase inhibitor	0.607	0.008
MELO3C010817.1	*CmSBT1.7b*	Subtilisin-like serine protease	−0.616	0.007

Furthermore, the expression patterns of *CmGoSAMTs* and *CmPMTs* members in ‘HDB’ and ‘HPM’ fruit at different developmental stages revealed that the *CmGoSAMT1*, *CmPMT1*, *CmPMT6*, and *CmPMT15* were the most abundantly expressed members in *CmGoSAMTs* and *CmPMTs* families, and there were significant difference between the two cultivars for all of these genes (Figs S7 and S8). However, the phylogenetic relationship exhibited that both *CmPMT1* and *CmPMT6* belong to Clade I and with similar expression pattern (Fig. S8), suggesting potential functional redundancy between them, while the *CmPMT1* has a higher mRNA transcriptional abundance level and a higher correlation coefficient with the DM of HG than *CmPMT6* (Fig. S6; Table 1). Therefore, *CmGoSAMT1*, *CmPMT1*, and *CmPMT15* were selected as target functional genes for further study.

### Overexpression of *CmPMT1* and *CmPMT15* makes fruit susceptible to softening, but not for *CmGoSAMT1*

**Figure 2:**
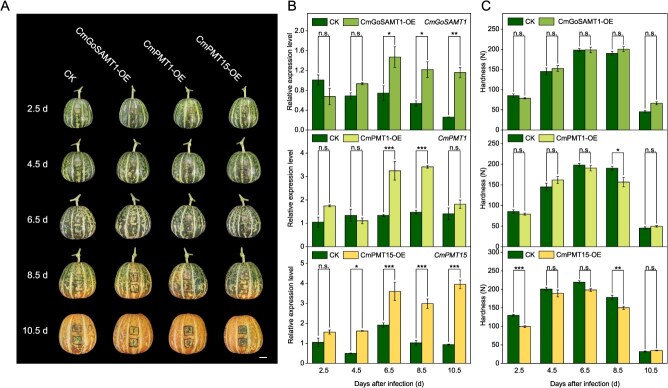
Effect on fruit hardness after overexpression of *CmGoSAMT1*, *CmPMT1*, and *CmPMT15*. (A) Fruit appearance and infection site after overexpression of *CmGoSAMT1*, *CmPMT1*, and *CmPMT15* in ‘HPM’ fruit. Scale bar = 2 cm. (B) Changes in gene expression level after overexpression of *CmGoSAMT1*, *CmPMT1*, and *CmPMT15*. (C) Changes in fruit hardness after overexpression of *CmGoSAMT1*, *CmPMT1*, and *CmPMT15*. Significant differences between the means were compared by Tukey test with ^*^P < 0.05, ^**^P < 0.01, and ^***^P < 0.001.

In order to identify the gene function of *CmGoSAMT1*, *CmPMT1*, and *CmPMT15,* transient overexpression using the CaMV 35S promoter was conducted in ‘HPM’ fruit. The results showed that the expression levels of the target genes in the sarcocarp tissue sites of *CmGoSAMT1*-OE, *CmPMT1*-OE, and *CmPMT15*-OE sites were gradually overexpressed from 2.5 DAI to 6.5 DAI and significantly higher than that of CK (over 2-fold) (Fig. 2B). Although, sarcocarp tissue hardness showed a consistent trend of increasing initially and then decreasing after infection, regardless of the sites of overexpression target genes or CK due to the defence response against the exogenous pathogen infestation of *Agrobacterium tumefaciens* in the experiment, which is with a suberification characteristics and an increased CWM content from 2.5 DAI to 6.5 DAI after infection (Fig. S9). However, at 8.5 DAI, the tissue hardness of *CmPMT1*-OE and *CmPMT15*-OE is significantly lower than that of CK, reaching *P* < 0.05 and *P* < 0.01 levels, respectively. But there was no significant change in hardness in *CmGoSAMT1*-OE tissue at 8.5 DAI. However, due to the large-scale degradation of CWM at the mature stage of fruit, there was no significant difference in hardness between CK and the other treatments at 10.5 DAI (Fig. 2C and Fig. S9).

### Overexpression of *CmPMT1* and *CmPMT15* reduces the extent of HG-Ca^2+^ cross-linking

**Figure 3:**
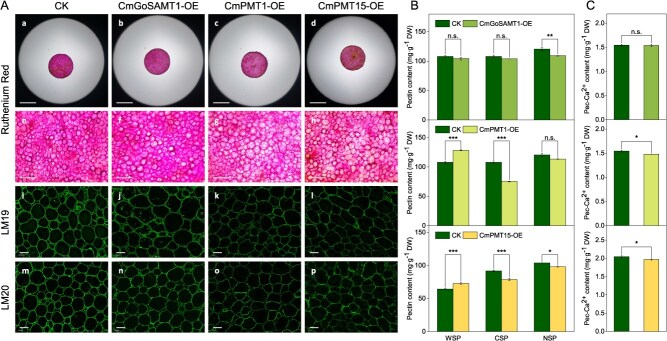
Effect on the abundance of de-methyl-esterified and methyl-esterified HG, pectin fractions content, and HG binding Ca^2+^ content after overexpression of *CmGoSAMT1*, *CmPMT1*, and *CmPMT15.* (A) Changes of de-methyl-esterified HG abundance represented by ruthenium red staining, and de-methyl-esterified and methyl-esterified HG abundance represented by immunofluorescence labelling using LM19 and LM20 monoclonal antibody at 8.5 days DAI. Scale bar = 1 cm for a–d, 500 μm for e–h, and100 μm for i–p. (B) Changes of pectin fractions content after overexpression of *CmGoSAMT1*, *CmPMT1*, and *CmPMT15* at 8.5 DAI. (C) Changes of HG binding Ca^2+^ content after overexpression of *CmGoSAMT1*, *CmPMT1*, and *CmPMT15* at 8.5 DAI. Significant differences between the means were compared by Tukey test with ^*^P<0.05, ^**^P<0.01, and ^***^P < 0.001.

Ruthenium red is a cationic dye that binds to carboxyl groups (acidic groups) via electrostatic binding and is commonly used to represent the abundance of de-methylated HG in the cell wall. Ruthenium red staining showed that the staining colour of *CmPMT1*-OE and *CmPMT15*-OE tissue sites was significantly lighter than that of CK, with the cell wall exhibited translucent, while the staining colour of *CmGoSAMT1*-OE tissue was just slightly lighter than that of CK (Fig. 3A-a to A-h). Meanwhile, the results of immunofluorescence labelling by LM19 and LM20 monoclonal antibodies showed that the fluorescence abundance of de-methyl-esterified HG of LM19 in *CmPMT1-OE* and *CmPMT15*-OE tissue sites was significantly weaker than that of CK, and the fluorescence abundance of LM19 in *CmGoSAMT1*-OE tissue showed slightly lower results than that of CK (Fig. 3A-i to A-l). For the LM20 monoclonal antibody for methyl-esterified HG, the results showed that the fluorescence abundance of LM20 in *CmPMT1*-OE and *CmPMT15*-OE tissue sites was also significantly weaker than that of CK, and that in *CmGoSAMT1*-OE tissue was also slightly lower than CK (Fig. 3A-m to A-p).

Furthermore, changes in pectin fractions (water-soluble pectin, WSP; chelator-soluble pectin, CSP; and Na_2_CO_3_-soluble pectin, NSP) and Pec-Ca^2+^ content in the cell wall of *CmGoSAMT1*-OE, *CmPMT1*-OE, and *CmPMT15*-OE tissue sites at 8.5 DAI showed that overexpression of *CmGoSAMT1* did not significantly alter the content of WSP, CSP, and Pec-Ca^2+^ content. However, overexpression of *CmPMT1* and *CmPMT15* both significantly increased the WSP content (*P* < 0.001) and significantly decreased CSP content (*P* < 0.001; Fig. 3B). Meantime, overexpression of *CmPMT1* and *CmPMT15* resulted in a significant decrease in Pec-Ca^2+^ content (*P* < 0.05; Fig. 3C). It is believed that the CSP is the de-methyl-esterified HG that cross-linked with Ca^2+^ in an ‘egg-box’ matrix form. Thus, the above results indicate that the overexpression of *CmPMT1* and *CmPMT15* resulted in a significant decrease in the amount of HG-Ca^2+^ cross-linked ‘egg-box’ matrix, whereas the overexpression of *CmGoSAMT1* had no significant effect on it. Meanwhile, the DM of HG all declined significantly after overexpression of *CmGoSAMT1*, *CmPMT1*, and *CmPMT15* at 8.5 DAI (Fig. S10). Finally, comprehensive analysis of the above results, we can deduce that overexpression of *CmPMT1* and *CmPMT15* not only alter the methyl-esterification of HG, but also leading to a large-scale degradation of HG, which caused by a reduction of HG-Ca^2+^ cross-linked ‘egg-box’ matrix in cell wall.

### Overexpression of *CmPMT1* and *CmPMT15* makes HG more accessible to *CmPMEs, CmPLs*, *and CmPGs*

The above experimental results indicated that the overexpression of *CmPMT1* and *CmPMT15* in ‘HPM’ fruit could make the HG in sarcocarp tissue more susceptible to degradation, but the underlying mechanism remains unclear. Therefore, the expression patterns of *CmPMEs*, *CmPGs*, and *CmPLs* were further analyzed based on the HDB_vs_HPM transcriptomes. Finally, three *CmPMEs* members *CmPME14* (MELO3C024306.1), *CmPME18* (MELO3C018374.1), and *CmPME36* (MELO3C005532.1), two *CmPGs* members *CmPG2* (MELO3C013129.1) and *CmPG37* (MELO3C011986.1), and one *CmPLs* member *CmPL13* (MELO3C012394.1) with higher expression abundance and showing significant difference in expression between the two cultivars were screened out (Fig. S11). Additionally, we noticed that *CmPL13* and *CmPME14* highly co-expressed with each other*,* and the expression patterns of *CmPG37* was similar to *CmPME18* and *CmPME36*, which suggested that there exists a relay mechanism among *CmPMEs, CmPLs*, and *CmPGs* in the process of HG de-methyl-esterification and degradation. Only *CmPG2* plays a key role in the dramatic softening at the mature stage of ‘HPM’ fruit (Fig. S12).

### CmPMT1 and CmPMT15 are capable for methyl transfer and assembly in structure

**Figure 4:**
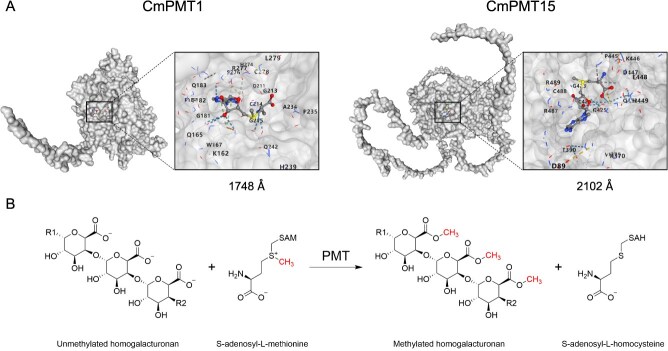
Molecular docking analysis of CmPMT1 and CmPMT15 with the ligand SAM. (A) Structure of the active pocket of the Methyltransf_29 functional domain of CmPMT1 and CmPMT15 binding to SAM. (B) Reaction process of unmethylated HG with methyl donor SAM under the catalytic action of PMT to generate methylated HG and release SAH.

Molecular docking analysis of CmPMT1 and CmPMT15 with the ligand SAM was performed based on the crystal structure predicted by AlphaFold. The cavity search results showed that there exist a 1748-Å and a 2102-Å pocket structure at the bottom of the Methyltransf_29 domain of CmPMT1 and CmPMT15, respectively (Fig. 4). Meanwhile, similar cavities were also found in the reported PMT proteins QUA2/TSD2/OSU1 (AT1G78240.1) and QUA3 (AT4G00740.1) in *A. thaliana*. Finally, the active amino acid residues interacting with SAM via hydrogen bonds in the above PMTs were analyzed according molecular docking. Thr182, Gly213, Gly215, Gln242, and Arg277 (sequencing based on CmPMT1) were identified as conserved active residues for binding to SAM in PMTs (Table S7).

**Figure 5:**
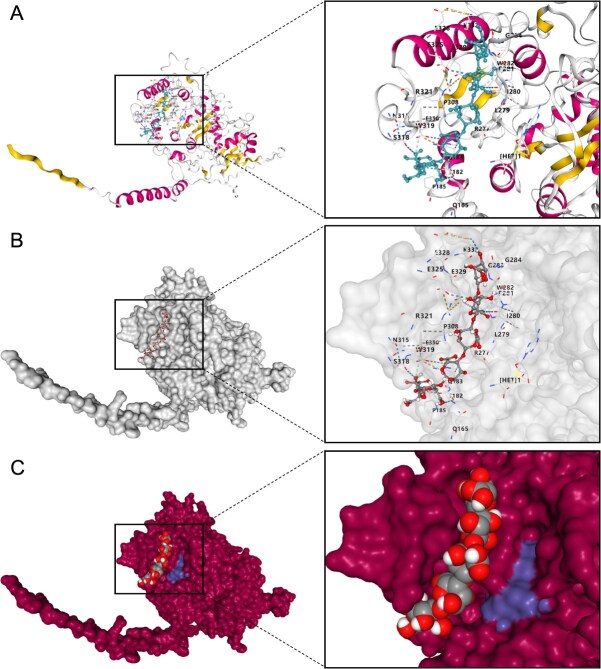
Molecular structure of the active binding pocket of CmPMT1. (A) Secondary structure of the pocket binding with a SAM and a 6-unit 2_1_-helix D-GalA chain. The α-helix is in carmine colour and the β-sheet is in yellow colour. (B) Surface structure of the pocket binding with a SAM and a 6-unit 2_1_-helix D-GalA chain. (C) Relative spatial position of the SAM to the 6-unit 2_1_-helix D-GalA chain in the pocket structure. The cavity area in purple is filled by the space of a SAM molecule.

In addition, the analysis of the secondary structure and surface structure of the active pocket of CmPMT1 showed that it has a ‘palm-like’ structure consisting mainly of a β-folded lamellae and α-helices above and along the side. Further docking revealed that the pocket also could also bind a 6-unit 2_1_-helix D-GalA HG chain. The amino acid residues Gln165, Thr182, Arg277, Gly283, Arg321, Glu325, Glu329, and Lys332 in the pocket exhibited hydrogen bond interactions with the 6-unit 2_1_-helix D-GalA HG chain may active residues (Fig. S16). Therefore, it was demonstrated that the pocket structure can catalyze the methyl group transfer and assembly to HG by capturing a SAM and binding to an HG chain simultaneously (Fig. 5).

### CmbZIP11 up-regulates *CmPMT1* and *CmPMT15* expression and affects fruit softening

**Figure 6:**
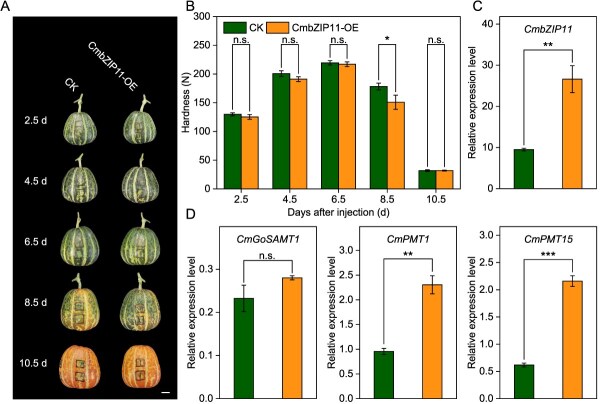
Effect on fruit hardness and expression of *CmGoSAMT1*, *CmPMT1*, and *CmPMT15* after overexpression of *CmbZIP11*. (A) Fruit appearance and infection site after overexpression of *CmbZIP11* in ‘HPM’ fruit. Scale bar = 2 cm. (B) Changes in fruit hardness after overexpression of *CmbZIP11*. (C) Changes in *CmbZIP11* expression level after overexpression at 8.5 days DAI. (D) Changes in gene expression level of *CmGoSAMT1*, *CmPMT1*, and *CmPMT15* after overexpression of *CmbZIP11* at 8.5 DAI. Significant differences between the means were compared by Tukey test with ^*^P < 0.05, ^**^P < 0.01, and ^***^P < 0.001.

Co-expression TFs with *CmGoSAMT1*, *CmPMT1* and *CmPMT15* were screened by WGCNA with correlation coefficient ≥ 0.9 (Fig. S17 and Table S6). Finally, we found that the overexpression of *CmbZIP11* (MELO3C005173.1) could up-regulate *CmPMT1* and *CmPMT15* expression and contribute fruit softening significantly at 8.5 DAI, and the significant level for *CmPMT1* and *CmPMT15* reached *P* < 0.01 and *P* < 0.001, respectively, but have no significant effect on the expression of *CmGoSAMT1* after overexpression of *CmbZIP11* (Fig. 6).

**Figure 7:**
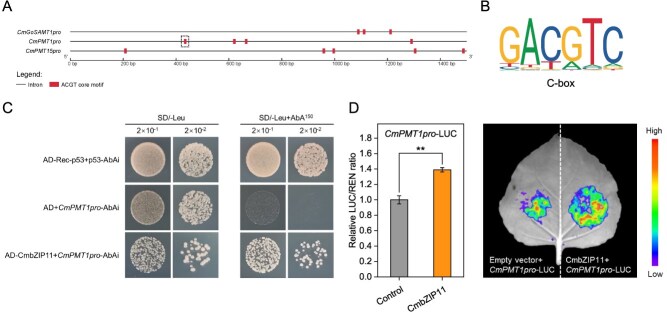
CmbZIP11 transcriptional activates the expression of *CmPMT1* by specifically binding to the C-box in the promoter region. (A) Analysis of the ACGT-core motif on *CmGoSAMT1*, *CmPMT1*, and *CmPMT15* promoter region. The boxed ACGT motif site on the *CmPMT1pro* is the C-box that the CmbZIP11 binds to. (B) Motif of C-box element. (C) Yeast one-hybrid assay (Y1H) verifies that CmbZIP11 binding to the C-box on the *CmPMT1* promoter. (D) Dual-luciferase reporter assay (Dual-LUC) demonstrates that CmbZIP11 transcriptionally activates the *CmPMT1* promoter expression. Significant differences between the means were compared by Tukey test with ^*^P < 0.05, ^**^P < 0.01, and ^***^P < 0.001.

Previous studies have reported that the bZIPs prefer to bind to *cis*-acting elements containing ACGT-core motif. Therefore, Y1H assays were conducted between CmbZIP11 with all promoter segments containing an ACGT-core motif of *CmGoSAMT1pro*, *CmPMT1 pro*, and *CmPMT15 pro* to examine the binding sites (Fig. 7A). It was finally demonstrated that CmbZIP11 could only bind to the C-box (GACGTC) on the *CmPMT1pro* at 1078–1073 bp upstream (Fig. 7B and C), but could not bind to any other motif. This may indicate that CmbZIP11 specifically recognizes the C-box. Furthermore, Dual-LUC reporter assay proved that CmbZIP11 could activate the expression of the *CmPMT1pro* (Fig. 7D). Additionally, it was demonstrated that CmbZIP11 is located in the cell nucleus (Fig. S19).

### Overexpression of *CmbZIP11* makes HG susceptible to degradation in fruit

**Figure 8:**
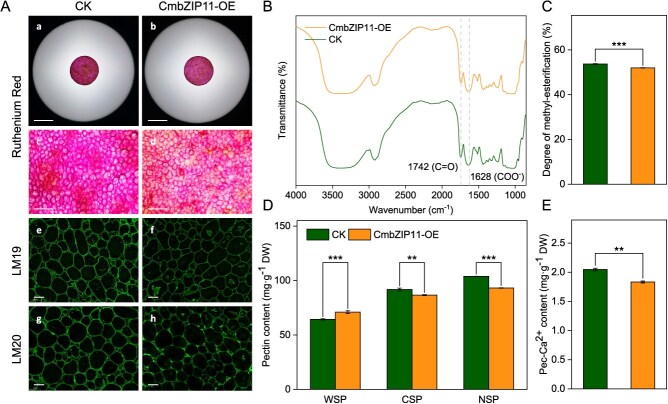
Effect on the abundance of de-methyl-esterified and methyl-esterified HG, degree of methyl-esterification of HG, pectin fractions content, and HG binding Ca^2+^ content after overexpression of *CmbZIP11* in ‘HPM’ fruit. (A) Changes of de-methyl-esterified HG abundance represented by ruthenium red staining, and de-methyl-esterified and methyl-esterified HG abundance represented by immunofluorescence labelling using LM19 and LM20 monoclonal antibody at 8.5 days DAI. Scale bar = 1 cm for a and b, 500 μm for c and d, and 100 μm for e–h. (B) FT-IR spectra transmittance curves of CWM range from 4000 to 850 cm^−1^ after overexpression of *CmbZIP11* and CK at 8.5 DAI. The characteristic absorption peaks of methyl ester C=O (1742 cm^−1^) and backbone COO^−^ (1628 cm^−1^) on HG are annotated by gray dashed lines. (C) Changes of degree of methyl-esterification of HG after overexpression of *CmbZIP11* at 8.5 DAI. (D) Changes of pectin fractions content after overexpression of *CmbZIP11* at 8.5 DAI. WSP: Water-soluble pectin; NSP: Na_2_CO_3_-soluble pectin; CSP: Chelator-soluble pectin. (E) Changes of HG binding Ca^2+^ content after overexpression of *CmbZIP11* at 8.5 DAI.

Overexpression of *CmbZIP11* at 8.5 DAI showed similar results to overexpression of *CmPMT1* and *CmPMT15*. The colour of ruthenium red staining of *CmbZIP11*-OE tissue was significantly lower than that of CK, and the abundance of immunofluorescent labelling of both de-methyl-esterified and methyl-esterified HG by LM19 and LM20 was significantly weaker than that of CK (Fig. 8A). The DM of HG decreased significantly (*P* < 0.001) (Fig. 8B and C). At the same time, the WSP content increased significantly (*P* < 0.001), and the CSP and NSP contents decreased significantly (*P* < 0.01 and *P* < 0.001, respectively) (Fig. 8D). Additionally, the Pec-Ca^2+^ content decreased significantly (*P* < 0.01) (Fig. 8E). In summary, the overexpression of *CmbZIP11* leads to a larger scale of degradation of HG relating to the up-regulated expression of *CmPMT1* and *CmPMT15.* This results in the synthesis of more highly methylated HG and causes a reduction in the amount of HG-Ca^2+^ cross-linked ‘egg-box’ matrix.

## Discussion

### HG methyl-esterification controlled by a complex spatial–temporal synergistic network

GS-MM screening of WGCNA showed that *CmGoSAMT1* and *CmPMT1*, *CmPMT6*, and *CmPMT15* highly correlated with the DM of HG (Table 1). Meanwhile, the expression patterns of *CmPMT1*, *CmPMT6*, and *CmPMT15* were similar in fruit, which indicated that the synergistic cooperation of multiple *CmPMTs* members is required in the process of methyl assembly of HG. Moreover, co-expression of *CmGoSAMT1* with *CmPMT1*, *CmPMT6*, and *CmPMT15* appears to ensure the spatial and temporal balance of supply and demand of the methyl donor SAM required for HG methylation (Figs S7 and S8).

Interestingly, *CmPMEs* members relating to de-methyl-esterification were not screened out by correlated DM of HG in this study, but a *CmSBTs* member (*CmSBT1.7b*) was (Table 1), which suggest that although PMEs are functional proteins that exercise de-methylation of HG, their ultimate activity *in vivo* may depend on SBTs, which determine the release of their active PME domains from the PRO region (PMEI domain) [17]. Therefore, it seems reasonable that the expression patterns of CmPME members do not significantly correlate with HG DM. In addition, it has been proven that the AtSBT3.5 can specifically recognize the processing motifs of AtPME17 in Arabidopsis [20]. At the same time, AtSBT3.3 and AtPME17 were found to be co-localized in the cell wall and to be highly co-expressed. Thus, the hypothesis that inactive Pro-PME17 in its unprocessed state is secreted into the cell wall with AtSBT3.3 at the same time and is subsequently processed by AtSBT3.3 to liberate its PME activity when needed [19]. However, an opposite result of increased PME activity and a de-methyl-esterified seed pectin mucilage phenotype were obtained in *atsbt1.7*, a knockout mutant of AtSBT1.7 [24]. Besides, the binding inhibition mechanism between PMEs and PMEIs complicates the spatiotemporal regulatory mechanism of active PMEs *in vivo*.

### CmPMTs rather than CmGoSAMTs are limiting factor in methyl assembly of HG

Currently, only a few putative PMTs have been investigated in plants, including QUA2/TSD2/OSU1 [25–29], QUA3 [30] in Arabidopsis, and homologous proteins in rice [31, 32]. It has been reported that PMTs located in the Golgi lumen catalyze the transfer of a methyl group from the donor SAM onto the *C*-6 carboxyl group of D-GalA residues of HG acceptor [14, 15]. However, only QUA2 has been shown indeed to have methyltransferase activity through *in vitro* experiments among them [28, 33].

Interestingly, the overexpression of *CmPMT1* and *CmPMT15* did not increase the DM of HG in sarcocarp tissue, but a decrease in the DM in this study. At the same time, we found that overexpressing of *CmPMT1* and *CmPMT15* made HG more susceptible to degradation, as evidenced by an increased WSP content, a decreased CSP and NSP content, and a decreased Pec-Ca^2+^ content, which leads to fruit prone to softening. The results of ruthenium red staining and immunofluorescent labelling with LM19/LM20 antibodies indicated de-methylated/methylated HG also confirmed this fact (Fig. 3). However, it is indisputable that PMTs perform methyl transfer and assembly functions during HG synthesis, since the results of molecular docking of CmPMT1 and CmPMT15 with a SAM and a 6-unit 2_1_-helix D-GalA HG chain suggest that the two proteins should possess the catalytic function in structure (Figs 4 and 5).

However, the overexpression of *CmGoSAMT1* did not significantly alter the fruit hardness or the abundance of HG in this study (Figs 2 and 3). Molecular structure of GoSAMT1 exhibits a ‘conical’ shape transmembrane channel consisting of 13 α-helical transmembrane domains, which demonstrated that CmGoSAMT1 act a bidirectional channel rather than a protein with catalytic activity (Fig. S13). On the other hand, it seems that the existing CmGoSAMTs have provided enough methyl donor SAM for the methyl assembly and it is CmPMTs, rather than CmGoSAMTs, that are the limiting factor in effecting the efficiency of HG methyl assembly. Therefore, overexpression of CmGoSAMT1 did not alter the degradation of HG and fruit hardness despite its high correlation with the DM of HG. However, Temple *et al*. found that the knockout mutations *gosamt1 gosamt2* of GoSAMTs in Arabidopsis exhibited reduced methylation of pectin and secondary cell wall xylan, and also stated that it is difficult to study the comprehensive role of pectin methyl esterification since the process of HG methylation is controlled by a range of enzymes, including numerous PMTs, PMEs, and PMEIs [16].

### CmPMTs determine the amount of HG-Ca^2+^ cross-linked ‘egg-box’ matrix altering accessibility for degradation

In this study, we further analyzed the expression patterns of *CmPMEs*, *CmPGs*, and *CmPLs* relating to HG de-methyl-esterification and degradation in two melon cultivars. Interestingly, we found there exit spatial–temporal co-expression between specific *CmPMEs* with *CmPGs* and *CmPLs* members, suggesting a ‘partner relationship’ between them, which ensured that HG can be cut down or degraded by *CmPLs* and *CmPGs* instantly after being de-methylated by *CmPMEs* but not to cross-link with Ca^2+^ to form an ‘egg-box’ matrix (Fig. S12).

Therefore, we propose a hypothesis that overexpression of *CmPMT1* and *CmPMT15* results in higher degree of methyl assembly of synthesized HG D-GalA in the Golgi apparatus and may even reach saturation. This makes HG to be transported to the cell wall in the form of a highly methylated free single chain rather than to cross-linking with Ca^2+^ to form the ‘egg-box’ matrix due to the ‘steric effect’ of the methyl group. Previous studies have illustrated that the ‘egg-box’ conformation of HG is tightly bound and structurally strong, making it difficult for PLs and PGs to access for hydrolyzing. In contrast, the highly methylated single chains HG free from each other with bigger interval space, making it easier to accesse by PMEs for de-methylation and further to hydrolyze by PLs and PGs subsequently for degradation [12, 34]. This hypothesis explains why HG is more susceptible to be degraded in the sarcocarp tissues after expression of *CmPMT1* and *CmPMT15* during softening. In addition, the DM of HG of sarcocarp tissues after overexpression of *CmGoSAMT1*, *CmPMT1*, and *CmPMT15* measured in this study may cannot represent the initial DM of HG after synthesis in the Golgi apparatus, since the HG will experience de-methylation by PMEs after being transported to the extracelullar space. Therefore, the function of CmGoSAMT1, CmPMT1, and CmPMT15 still needs further validation using a eukaryotic expression experiment *in vitro*.

### CmbZIP11 is a key TF regulating HG methyl-esterification

Basic/region leucine zippers (bZIPs) factors consist of a bZIP conserved structural domain of 60–80 amino acids (aa), divided into an N-terminal basic region and a C-terminal leucine ZIP region. The basic region is relatively conserved and contains 16 aa residues that are responsible for nuclear localization and DNA binding (N-X_7_-R/K-X_9_), whereas the leucine ZIP region is a 7-peptide repeat region (L-X_6_-L-X_6_-L) that is not conserved and responsible for controlling homodimerization/heterodimerization of bZIPs [35, 36]. It has been reported that the basic region of bZIPs preferentially bind to ACGT-core palindromic motif, such as A-box (TACGTA), C-box (GACGTC), and G-box (CACGTG) [37]. In Arabidopsis, 78 bZIPs members were identified, which were classified into 13 groups (A–M) based on phylogenetic relationship [35, 36], whereas 75 CmbZIPs were identified in melon [38]. Based on the homology with Arabidopsis bZIPs members, the CmbZIP11 in this study belongs to Group S.

In this study, overexpression of *CmbZIP11* significantly up-regulated the expression of *CmPMT1* and *CmPMT15* (*P* < 0.01 and *P* < 0.001, respectively), as well as the changes in fruit hardness, DM of HG, ruthenium red staining, immunofluorescent labelling of de-methyl-esterified and methyl-esterified HG using LM19 and LM20 antibodies, the content of pectin fractions, and Pec-Ca^2+^ content after overexpression of *CmbZIP11* were all consistent with the results obtained after *CmPMT1* and *CmPMT15* overexpression (Figs 6 and 8). Furthermore, Y1H and Dual-LUC assays demonstrated that CmbZIP11 can directly bind to the C-box element on the *CmPMT1pro* to activate its expression. Meanwhile, although overexpression of *CmbZIP11* also significantly induced the expression of *CmPMT15*, there was no C-box element on the promoter region of *CmPMT15* (Fig. 7). Therefore, it is speculated that CmbZIP11 may indirectly regulate the expression of *CmPMT15* through cascading or by interacting with other TFs. In addition, we plan to obtain CmbZIP11 mutant melon plants and fruits via CRISPR-Cas9 gene editing to further confirm its function in regulating the methyl-esterification of HG and fruit softening in melon.

## Conclusions

**Figure 9:**
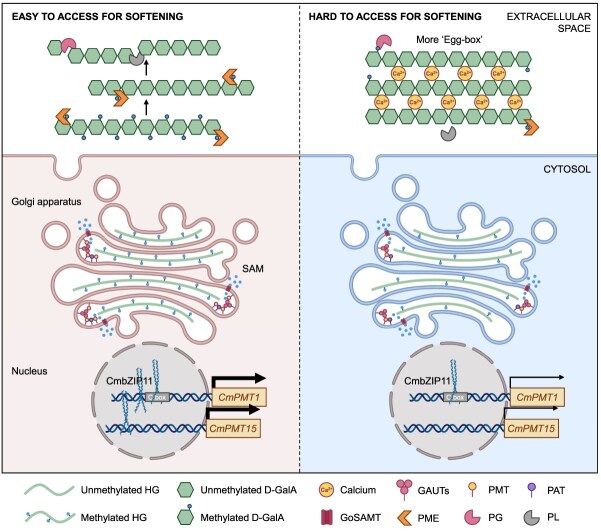
Mechanism of CmbZIP11 regulates *CmPMT1* and *CmPMT15* expression to regulate methyl-esterification of HG affecting fruit softening in melon. CmbZIP11 through activates the expression of *CmPMT1* and *CmPMT15* to regulate the degree of methyl-esterification of HG thereby adjust the amount of HG-Ca2+ cross-linked `Egg-box' matrix, leading to different accessibility for PMEs, PGs and PLs for HG degradation and fruit softening. HG: Homogalacturonan; D-GalA: D-Galacturonic acid; GAUTs: Galacturonosyltransferases; GoSAMT: Golgi SAM transporter; PMT: Pectin methyltransferase; PAT: Pectin acetyltransferase; PME: Pectin methylesterase; PG: Polygalacturonase; PL: Pectate lyase.

It was hypothesized that the high-level expression of *CmPMT1* and *CmPMT15* resulted in HG existing predominantly in the form of highly methylated free chains due to the ‘steric effect’ of methyl group on the *C*-6 carboxyl of the D-GalA residue of HG, thereby can not be cross-linked by Ca^2+^ to form ‘egg-box’ matrix, making it easy to access for CmPMEs to de-methylation and to be cutdown or degraded by CmPLs and CmPGs successively, thus making the fruit susceptible to softening. Conversely, under low-level expression of *CmPMT1* and *CmPMT15*, synthesized low-methylated HG with more exposed anions at the *C*-6 carboxyl prone to cross-link with Ca^2+^ to form ‘egg-box’ matrix, which is hard to access for CmPMEs, CmPLs, and CmPGs, thus blunt to softening. Molecular docking analysis based on AlphaFold prediction revealed there exist a 1748-Å and a 2102-Å ‘palm-like’ pocket at the bottom of the Methyltransf_29 domain of CmPMT1 and CmPMT15, respectively. The pocket can capture a methyl donor SAM and can also bind an HG chain of 6-unit 2_1_-helix D-GalA, should exercise a catalytic function in methyl transfer and assembly. Additionally, CmbZIP11 was found to play a role in regulating the HG methylation by up-regulating the expression of *CmPMT1* and *CmPMT15* (Fig. 9).

## Materials and methods

### Plant materials

Two oriental melon (*C. melo* var. *makuwa* Makino) cultivars ‘Hongdaobian’ (Kaifeng Zhongbo Seedling Research Institute, China) with crisp texture fruit and ‘Hongpimian’ (Hebei Baoding Seedling Company, China) with mealy texture fruit were taken as materials, which are abbreviated as ‘HDB’ and ‘HPM’, respectively. The plants were grown in substrate bags in a greenhouse at Shenyang Agricultural University in Shenyang, Liaoning Province, China. Single stem training was used, and each plant was set with three fruits from the tenth node. Fruits from the same node that did not have any disease, insect pests, or mechanical injury were chosen for sampling. Furthermore, three biological replicates were set at each stage. The fruits were sampled according to days after anthesis (DAA): the ‘HDB’ fruits were sampled at 15 DAA, 25 DAA, and 35 DAA and the ‘HPM’ fruits were sampled at 15 DAA, 22 DAA, and 32 DAA corresponding to the fruitlet, expanding, and mature stages of fruit development, respectively (Fig. S1). The sarcocarp from the equatorial part of the fruit was sampled, frozen with liquid nitrogen, and stored at −80°C.

### RNA-seq and analysis

Samples of ‘HDB’ and ‘HPM’ fruits at the fruitlet, expanding, and mature stages were used to construct the transcriptome library. Qualified RNA extracted using the CTAB method to construct the cDNA libraries, which were then sequenced on an Illumina platform (NovaSeq 6000 System, USA) at Metware Biotechnology Co., Ltd. (Wuhan, China). The mapped efficiency of the RNA-seq data against the melon genome Melon (DHL92) v4 (http://cucurbitgenomics.org/v2) using Hisat2 ranged from 96.61% to 97.44% [39]. The raw sequencing data are available on the NCBI SRA (https://www.ncbi.nlm.nih.gov/sra) with the BioProject accession number of PRJNA1249191.

WGCNA and GS-MM analysis was performed using the Metware Cloud online platform (https://cloud.metware.cn). First, the varFilter function in genefilter package of the R language was used to remove the genes showing low expression or with constant expression in all samples. Then, the WGCNA was performed with the *R*^2^ cutoff set as 0.85 and the module merge cut height set as 0.25. The correlation between fruit hardness and DM of HG and modules was analyzed. The gene co-expression network of candidate TFs with target functional genes was visualized using the igraph tool in Hiplot Pro (https://hiplot.com.cn).

### Identification of HG methyl-esterified modification related gene families in melon

Firstly, the melon genome Melon (DHL92) v4 was downloaded from the Cucurbit Genomics Database v2 (CuGenDBv2; http://cucurbitgenomics.org/v2). Then, protein sequences against Pfam conserved domain for CmGoSAMTs (MFS_5: PF05631), CmPMTs (Methyltransf_29: PF03141), CmPMEs (Pectinesterase: PF01095), CmPMEIs (PMEI: PF04043), and CmSBTs (Peptidase_S8: PF00082) were searched genome-wide using the Advanced HMMer Search plugin in TBtools-II software [40]. Finally, the obtained protein sequences were further examined via Simple Modular Architecture Research Tool (SMART; http://smart.embl-heidelberg.de). Phylogenetic trees were constructed by MEGA 11 software after multiple sequence alignment [41].

### Gene transient overexpression in melon fruit

The coding sequences (CDS) of *CmGoSAMT1*, *CmPMT1*, and *CmPMT15* were cloned and inserted into the pCAMBIA3301-LUC vector to construct the *35S:CmGoSAMT1*-LUC, *35S:CmPMT1*-LUC, and *35S:CmPMT15*-LUC recombinant vectors. These recombinant vectors were then transformed into *A. tumefaciens* EHA105 competent cells using the freeze–thaw method. Transient gene overexpression in melon fruit was performed as follows: firstly, the transformed competent cells of *35S:CmGoSAMT1*-LUC, *35S:CmPMT1*-LUC, and *35S:CmPMT15*-LUC vectors were amplified using YEP liquid medium (containing 50 mg·L^−1^ kanamycin and 25 mg·L^−1^ rifampicin) under 28°C at 180 rpm until the bacterial concentration reached OD_600_ = 1.0–1.2. The bacteria were then collected and washed twice with sterilized water and resuspended with the infiltration buffer [10 mM 2-(N-morpholino) ethanesulfonic acid+10 mM MgCl_2_·6H_2_O + 200 μM Acetosyringone + 0.005% Tween 20 (v/v)], and stored in the dark at 28°C for 3 h. Finally, the infiltration buffer was injected into the sarcocarp of ‘HPM’ fruits at 22 DAA using sterilized syringes with 0.5 ml for each injection site. The empty vector was used as a control. Sarcocarp tissue from the infiltrated sits were sampled using a puncher (1.5 cm φ) at 2.5, 4.5, 6.5, 8.5, and 10.5 days after infection (DAI), respectively. Additionally, slices of sarcocarp approximately 1 mm thick from the infection sites were immersed in 1 mM-1 D-Luciferin potassium (MCE HY-12591B, USA; containing 0.1% Tween 20),and left to react in the dark for 10 min. Finally, LUC signal was detected using an *in vivo* plant fluorescence imaging system (Berthold NightSHADE LB 985, Germany) to verify if it transformed successfully (Fig. S18). Five biological replicates for each treatment, with each fruit injected at two different sites near the equator. Relative primers are listed in Tables S4 and S5.

### RNA isolation and RT-qPCR

Total RNA was extracted using an ultrapure RNA kit (CWBIO CW0581M, China) and was then reverse-transcribed into cDNA using a Primer Script RT reagent kit (TaKaRa PrimeScript™ RT Master Mix, Japan). RT-qPCR reactions were performed using TransStart Top Green qPCR SuperMix (TransGen Biotech, China) on a Real-Time PCR Thermal Cycler (Analytic Jena AG qTOWER^3^ G, Germany). The following is the PCR programme: initial denaturation at 95°C for 30 s, followed by 40 cycles of 95°C for 5 s and 60°C for 34 s, then melt for 15 s. A *C. melo* ribosomal RNA gene (*18S*) was used as an endogenous control for normalization. Gene relative expression was calculated using the 2^−ΔΔCt^ method [42]. Each sample was analyzed in triplicate. The specific primers, which were for RT-qPCR designed by the PrimerQuest Tool (https://sg.idtdna.com/PrimerQuest/Home/Index), are listed in Table S3.

### Fruit hardness

The fruit hardness detection method was adapted from Pan *et al*. [23]. Column-shaped sarcocarp samples with 1.5-cm diameter and 1-cm height were obtained from the infiltration sites after overexpression of target genes by a puncher. Hardness was then determined under TPA (textural profile analysis) mode using a texture analyzer (TMS-Pilot, FTC, USA) with a 75-mm platen using trigger point force as 0.75 N, test speed as 30 mm/min, and deformation variable as 30%. Ten technical replicates were set for each treatment.

### Cell wall materials isolation

The method referred to Rose *et al*. [43]. Firstly, 15 g of sarcocarp was homogenized with 95% ethanol and fixed the volume to 30-ml bath at 90°C for 0.5 h to passivate the cell wall enzymes. Then, it was extracted twice with 80% ethanol at 90°C to remove sugar and pigment. Next, the residues were extracted with 30 ml of 90% dimethyl sulfoxide at 4°C overnight to remove starch. After that, it was extracted with 30 ml of chloroform:methanol (1:1, v/v) at 25°C for 4 h, followed by 30 ml of acetone at 25°C for 4 h subsequently to get rid of any protein or fat. Finally, the cell wall material (CWM) residues were collected and dried at 45°C for 12 h.

### FT-IR spectroscopy and degree of methyl-esterification calculation

The method referred to Pan *et al*. [23]. A total of 5 mg of dried CWM was mixed with spectroscopic-grade potassium bromide (1:100, w/w), ground into powder and then compacted into discs using a tablet machine (Tianjin Tuopu FW-4A-1, China). The FT-IR spectra of CWM was measured using a FT-IR spectrometer (Thermo Scientific Nicolet iS50 FT-IR, USA). Each disc was scanned 100 times over the wavenumber range from 4000 cm^−1^ to 400 cm^−1^ at a resolution of 4 cm^−1^ under absorbance mode. The calculation of DM of HG referred to Kyomugasho *et al*. [44]. The absorbance of C=O stretching vibration of alkyl ester (1745–1740 cm^−1^) and COO^−^ antisymmetric stretching (1630–1625 cm^−1^) were read as *A_1_* and *A_2_*, respectively.


(1) \begin{equation*} \mathrm{HG}\ \mathrm{DM}\ {\textrm (}\%{\textrm )}=1.0405\times \mathrm{FT}{\textrm -}\mathrm{IR}-\mathrm{IR}\ \mathrm{DM}\ {\textrm (}\%{\textrm )}-3.9096. \end{equation*}

(2) \begin{equation*} \mathrm{FT}{\textrm -}\mathrm{IR}\ \mathrm{DM}\ {\textrm (}\%{\textrm )}=136.86\times R+3.987. \end{equation*}

(3) \begin{equation*} R={A}_1/{\textrm (}{A}_1+{A}_2{\textrm )}. \end{equation*}

### Ruthenium red staining

Fruit sarcocarp discs with a diameter of 1.5 cm and thickness of 1 mm were cut using a blade, immersed into 0.05% ruthenium red staining solution, infiltrated under vacuum for 3 min, then washed with sterilized water for 3 min. Finally, the stained discs were observed and photographed using a stereomicroscope (Nikon SMZ 745T, Japan). Three replicates were set for each treatment.

### Immunofluorescence labelling

Immunofluorescence labelling of paraffin sections was performed according to the method described by Pan *et al*. [23]. The LM19 (Agrisera AS18 4191, Sweden) and LM20 antibodies (Agrisera AS18 4193, Sweden) against de-methyl-esterified or methyl-esterified HG were used. Fluorescent labelling was observed using a fluorescence microscope (Nikon ECLIPSE C1, Japan) under FITC excitation wavelengths of 465–495 nm and emission wavelengths of 515–555 nm (green).

### Pectin fractions extraction and determination

The pectin fraction extraction method was adapted from Santiago-Doménech *et al*. [45]. WSP, CSP, and NSP were extracted from 50 mg of CWM sequentially with 10 ml of distilled water, 50 mM CDTA solution (containing 50 mM^−1^ sodium acetate, pH = 6.5), and 50 mM Na_2_CO_3_ solution (containing 20 mM NaBH_4_) by shaking at 25°C with 200 rpm for 4 h for each fraction. The uronic acid content in the different fractions was determined by the carbazole-sulphuric acid colourimetric method [46] using a microplate reader (TECAN Spark®, Switzerland) with three technical replicates.

### Pectin binding Ca^2+^ extraction and determination

The extraction method for pectin-bound Ca^2+^ referred to 小西 and 葛西 [47]. Pectin-bound Ca^2+^ (Pec-Ca^2+^) fraction was extracted from 50 mg of dried CWM with 10 ml of 1 M NaCl solution at 25°C and 200 rpm for 4 h with oscillation after the water-soluble Ca^2+^ fraction had been extracted with 10 ml of deionized water first. The filtrate was collected by filtering through a 0.22-μm aqueous membrane. The Ca^2+^ content was determined by an atomic absorption spectrometry (Thermo Scientific iCE 3000 GF AAS, USA) with three technical replicates.

### Promoter TFs binding site analysis

The 1500-bp promoter sequences upstream of *CmGoSAMT1*, *CmPMT1*, and *CmPMT15* were extracted from melon genome Melon (DHL92) v4 using the Gtf/Gff3 Sequences Extract function in TBtools-II software [40]. TFs binding sites on the promoter sequences were predicted using the JASPAR (https://jaspar.elixir.no) and the PlantPegMap (https://plantregmap.gao-lab.org/index.php) websites. Gene Structure Display Server (GSDS) 2.0 was used to visualize the TFs binding sites [48].

### Yeast one-hybrid assay

The CDS of *CmbZIP11* was inserted into the pGADT7 vector to construct the CmbZIP11-AD prey vector. Promoter segments about 200 bp containing the ATCG-core motif from the *CmGoSAMT1pro*, *CmPMT1pro*, and *CmPMT15pro* were inserted into the pAbAi vector as baits. The bait plasmid was then linearized and transformed into Y1H Gold Yeast using a kit (Takara YeastmakerTM Yeast Transformation System 2, Japan). The strains were then screened on SD/-Ura medium containing gradients of Aureobasidin A (AbA; 0, 50, 75, 100, 150, 200, and 300 ng·ml^−1^). Finally, the interaction between CmbZIP11 and the promoter segments was examined on SD/-Leu medium at the optimum AbA concentration after cultivation at 30°C for 3 d. The strains co-transformed with the empty pGADT7 vector with the corresponding recombinant pAbAi vectors were used as negative control, and the strains co-transformed with pGADT7-Rec-p53 and p53-AbAi were used as positive control. The vector primers are listed in Table S5.

### Dual-luciferase reporter assay

The *CmPMT1pro* sequence was inserted into the pGreenII 0800-LUC vector as a reporter and then transformed into *A. tumefaciens* GV3101 (pSoup) competent cell. The CmbZIP11-pCAMBIA1300-eGFP recombinant vector was used as effector and transformed into GV3101 competent cell. The *A. tumefaciens* infiltration buffer containing *CmPMT1pro*-pGreenII0800-LUC and CmbZIP11-pCAMBIA1300-eGFP recombinant vectors was mixed at a ratio of 1:9 (v/v). The pCAMBIA1300-eGFP empty vector was used as the control. The infiltration buffer was then injected into the lower epidermis of *Nicotiana benthamiana* leaves using a 1-ml syringe without needle. The tobacco plants were then cultivated in the dark for 1 day and subsequently moved to light for 2 days. Finally, the infiltrated tobacco leaves were picked and sprayed with 1 mM^−1^ D-Luciferin potassium (MCE HY-12591B, USA) containing 0.1% Tween 20. LUC signal was detected using a plant *in vivo* fluorescence imaging system (Berthold NightSHADE LB 985, Germany). The activities of LUC and REN were detected using a Dual-LUC reporter assay kit (TransGen Biotech FR201, China) with three technical replicates.

### Subcellular location

The CmbZIP11-pCAMBIA1300-eGFP recombinant vector was transformed into *A. tumefaciens* GV3101 competent cells for the infiltration of *N. benthamiana* leaves. Fluorescence in the dorsal epidermal cells of the tobacco leaves was captured 3 days after infiltration using confocal laser scanning microscopy (ZEISS LSM 900, Germany), and 4′,6-diamidino-2-phenylindole (DAPI) (SL7100 Coolaber, China) was used as cell nucleus localization marker. Excitation/emission wavelengths were 488 nm/507 nm and 364 nm/454 nm for eGFP and DAPI, respectively.

### Molecular docking simulation

The protein crystal structures of CmGoSAMT1 (AF-A0A1S3AV21-F1), CmPMT1 (AF-A0A1S3B421-F1), and CmPMT15 (AF-A0A5D3C6E9-F1) predicted by AlphaFold were downloaded via UniProt (https://www.uniprot.org). The three-dimensional structures that were visualized using SWISS-MODEL (https://swissmodel.expasy.org) are showed as Figs S13 and S14. The Ramachandran plot of CmGoSAMT1, CmPMT1, and CmPMT15 protein modelling structures are shown in Fig. S15. The ligand structure files of SAM and HG were downloaded from PubChem (https://pubchem.ncbi.nlm.nih.gov), and the protein-ligand molecular docking simulation were performed using the online tool CD-Dock2 [49]. The protein-ligand interaction hydrogen bonding was analyzed and visualized using PyMol V2.6.0a0 (Schrödinger LLC, USA) and LigPlus [50].

### Statistical analysis

Microsoft Excel 365 was used to process the raw data. Significant differences in means were compared by Tukey test via the Paired Comparison Plot App in OriginPro 2021 software (OriginLab Corporation, USA). Heatmaps of gene expression were drawn by TBtools-II software after the FPKM values were log_2_-transformed [40]. Lastly, the chemical formula was created using ChemDraw V 23.1.1.3 (Revvity, USA).

## Accession numbers

Gene ID of *CmGoSAMTs*, *CmPMTs*, *CmPMEs*, *CmPMEIs*, and *CmSBTs* members of *Cucumis melo* from CuGenDB (http://cucurbitgenomics.org/v2) are provided in Table S1. Gene ID of *AtGoSAMTs* and *AtCmPMTs* members of *Arabidopsis thaliana* from TAIR (http://www.arabidopsis.org) are provided in Table S2.

## Supplementary Material

Web_Material_uhaf253
