# Supplementary material for: CmbZIP11 regulates CmPMT1/15 affecting homogalacturonan methyl-esterification and fruit softening in melon
Source: Hortic Res. 2025 Sep 12;13(1):uhaf253. doi: 10.1093/hr/uhaf253 (PMC12881853; doi:10.1093/hr/uhaf253)
Supplement: Web_Material_uhaf253 [file web_material_uhaf253.zip › Supplementary Figure.docx]

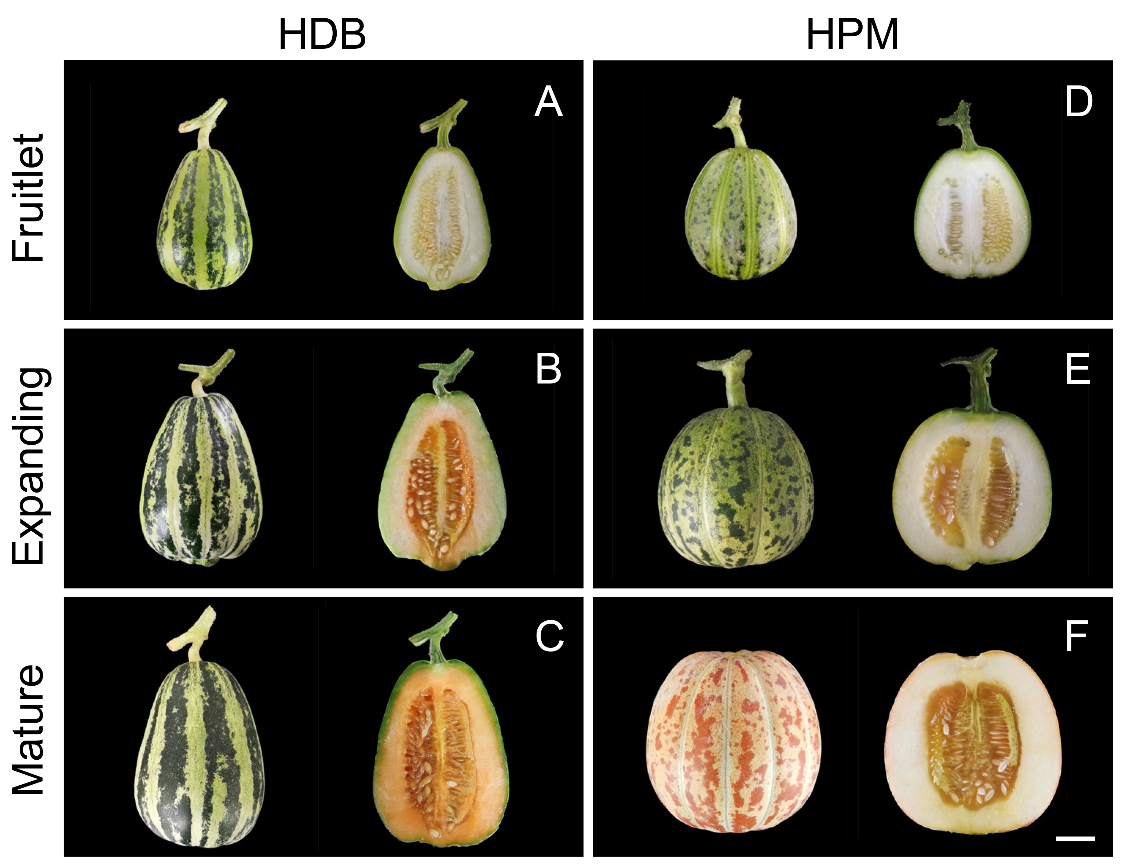


**Figure S1.** Fruit appearance of ‘HDB’ and ‘HPM’ at different developmental stages.

Fruitlet stage: 15 DAA; Expanding stage: 25 DAA; Mature stage: 35 DAA or 32 DAA for ‘HDB’ and ‘HPM’, respectively. DAA: Days after anthesis. Scale bar = 2 cm.


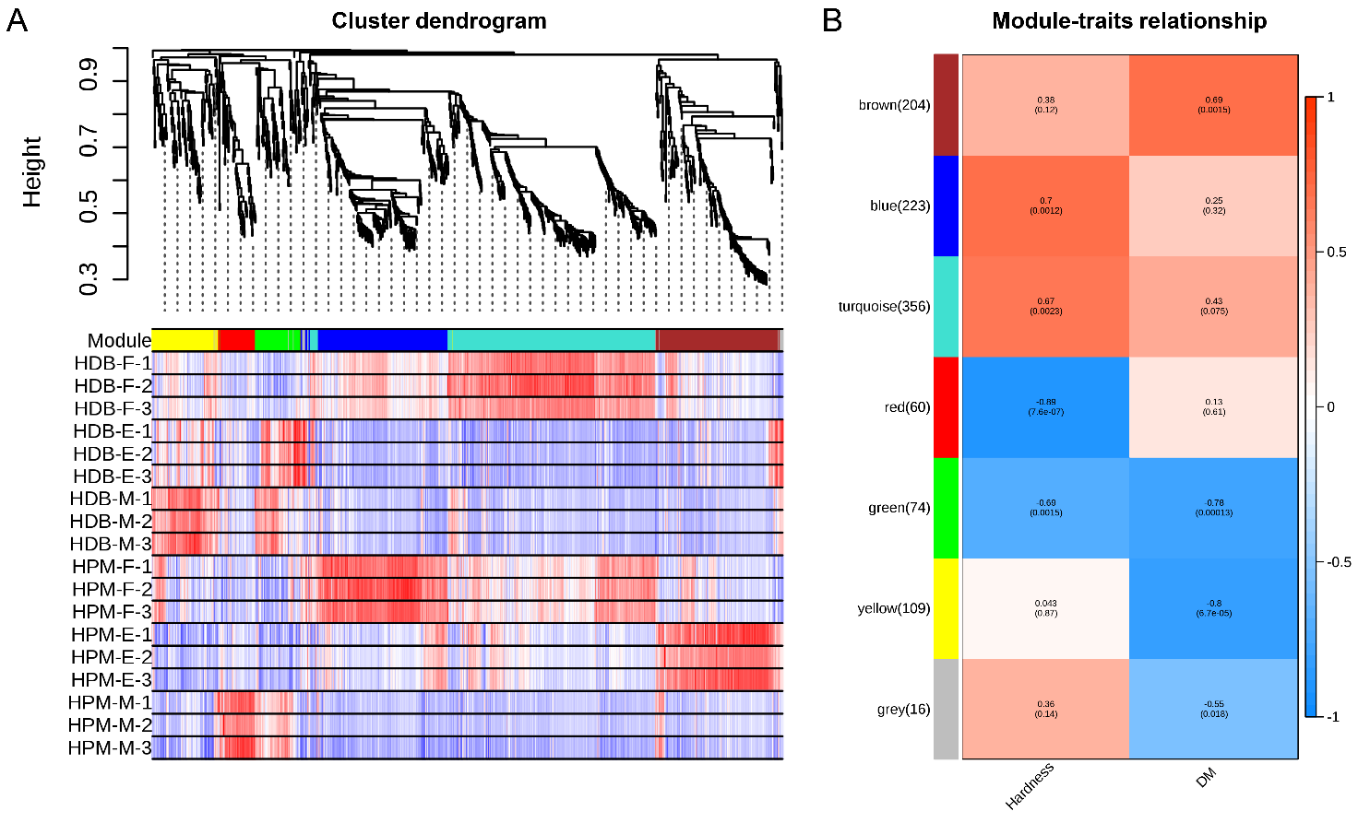


**Figure S2.** Weighted gene co-expression network analysis of HG methyl-esterification related genes in the HDB_vs_HPM transcriptome. **A)** Hierarchical clustering of gene modules expression; **B)** Correlation analysis between modules with hardness and degree of methyl-esterification of HG.


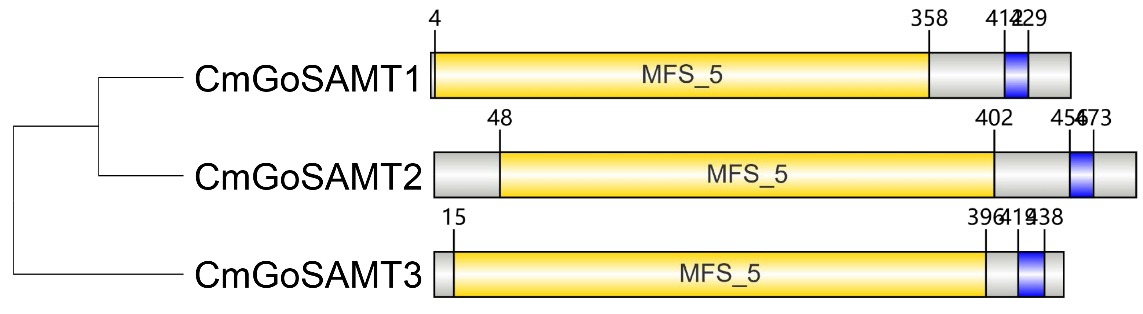


**Figure S3.** Conserved functional domains distribution of CmGoSAMTs. The yellow box represents MFS_5 domain (PF05631), the bule box represents transmembrane domain.


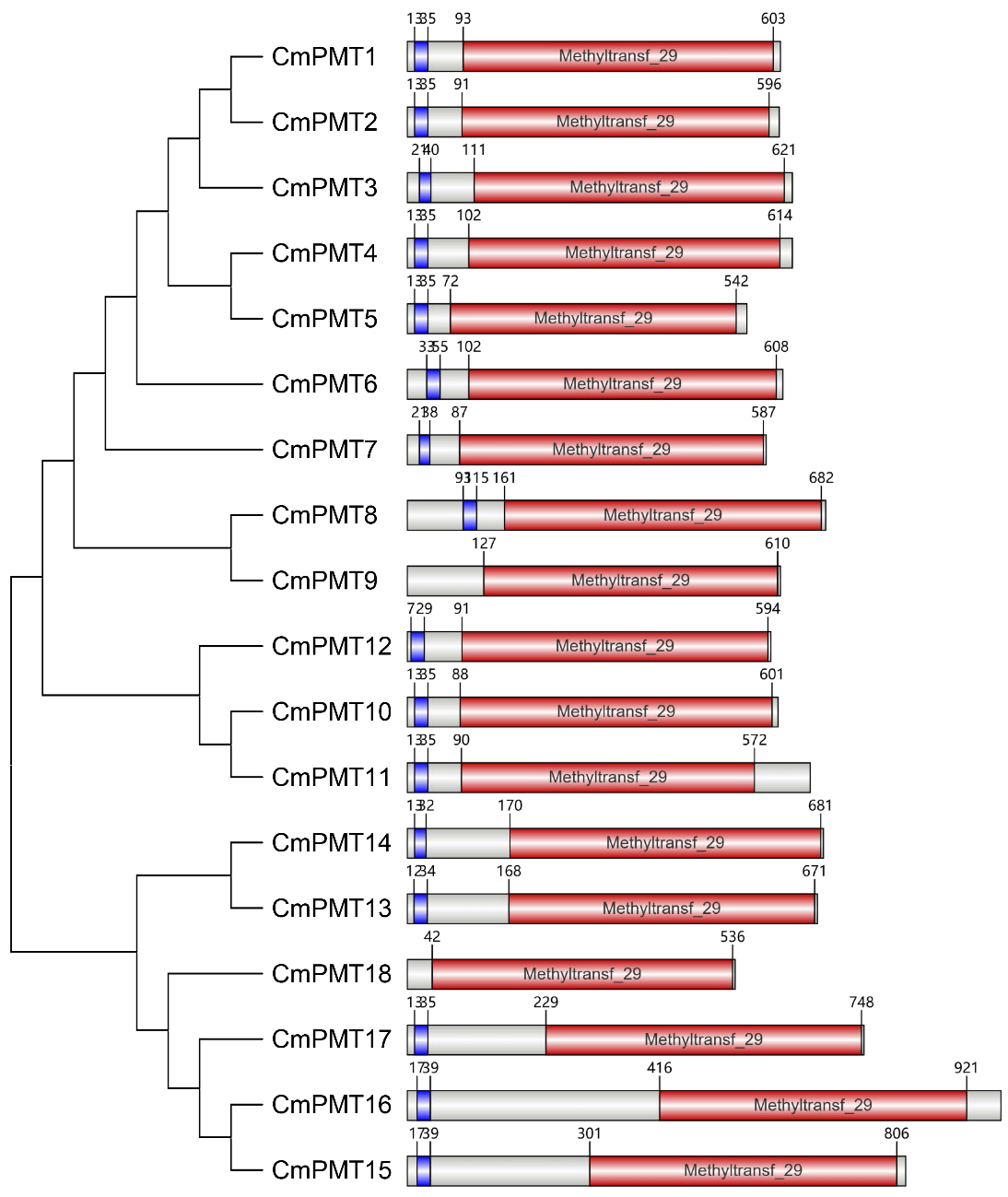


**Figure S4.** Conserved functional domains distribution of CmPMTs. The red box represents Methyltransf_29 domain (PF03141), the bule box represents transmembrane domain.


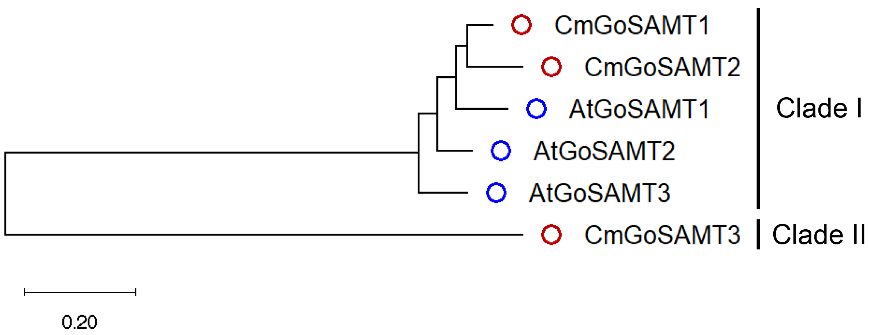


**Figure S5.** Phylogenetic tree of GoSAMTs among *Cucumis melo* and *Arabidopsis thaliana* members.


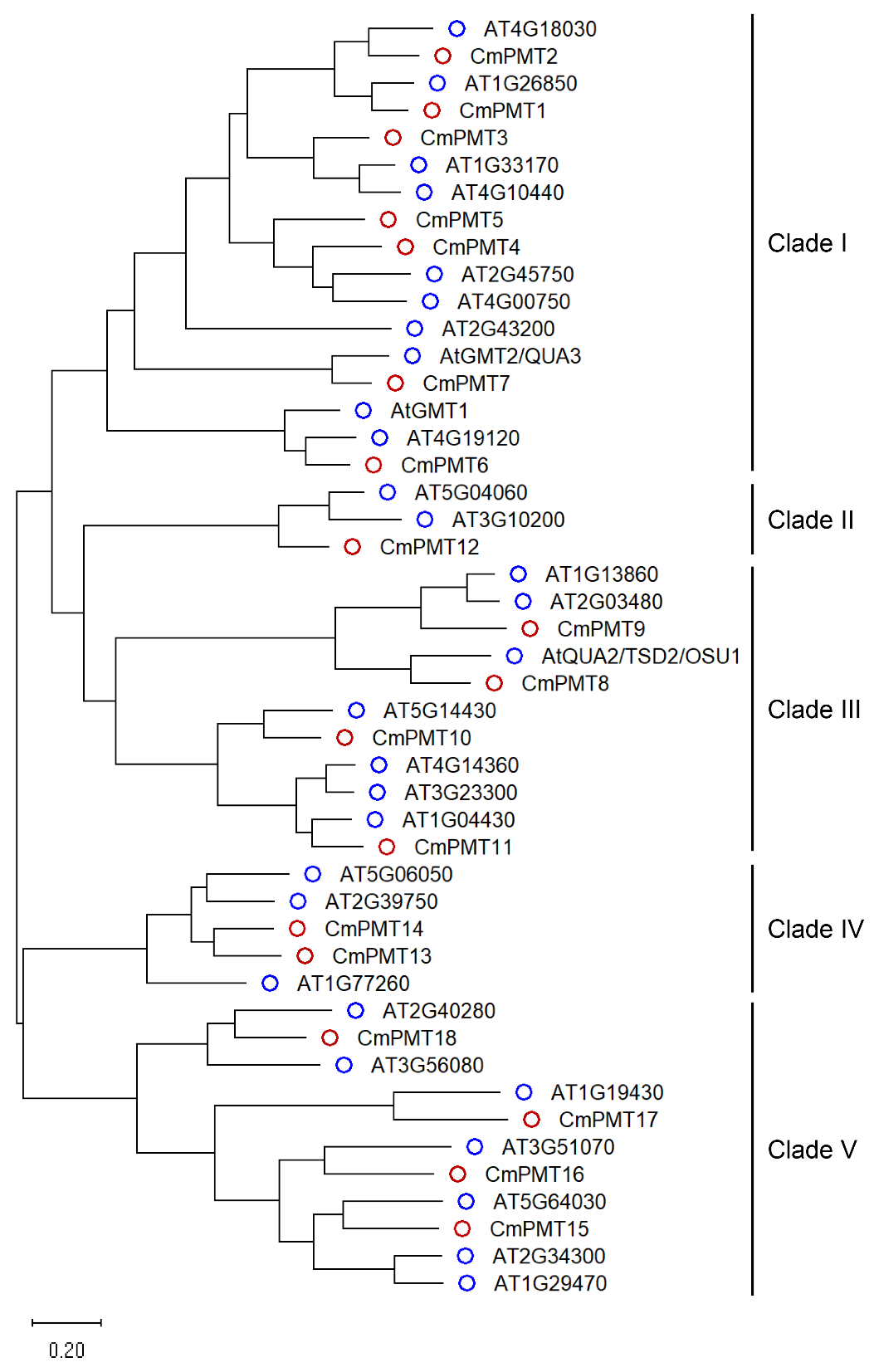


**Figure S6.** Phylogenetic tree of PMTs among *Cucumis melo* and *Arabidopsis thaliana* members.


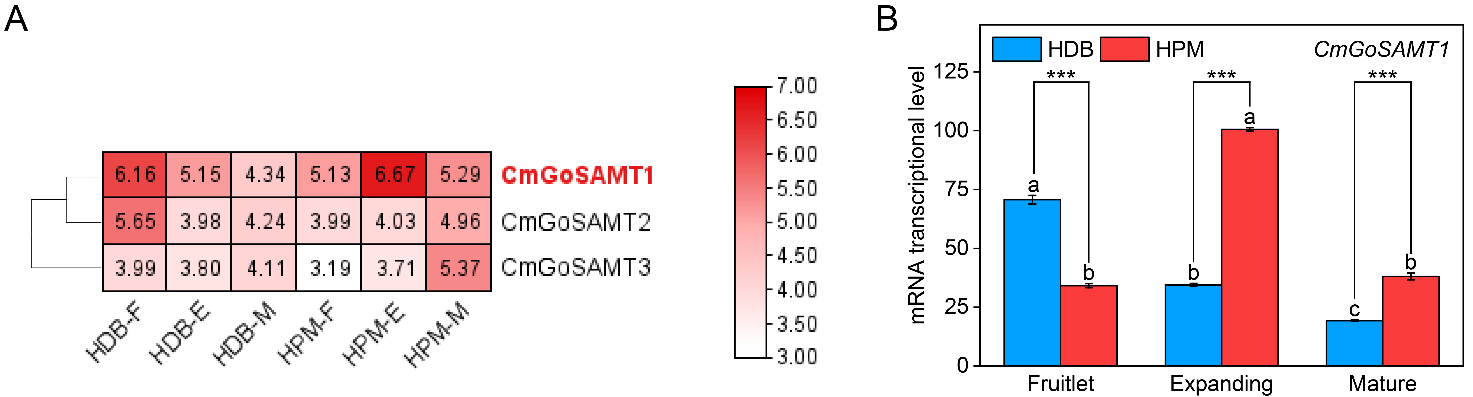


**Figure S7.** Gene expression pattern of *CmGoSAMTs* during ‘HDB’ and ‘HPM’ fruit development. **A)** Heatmap of *CmGoSAMTs* expression pattern. The gene expression of FPKM were log_2_-transformed. **B)** Differential analysis of *CmGoSAMT1* expression in ‘HDB’ and ‘HPM’ fruit during development. Significant differences between the means were compared by Tukey test with * *P* < 0.05, ** *P* < 0.01 and *** *P* < 0.001. Different letters on each column indicate significant differences between the means among different developmental stages within each cultivar at *P* < 0.05 level.


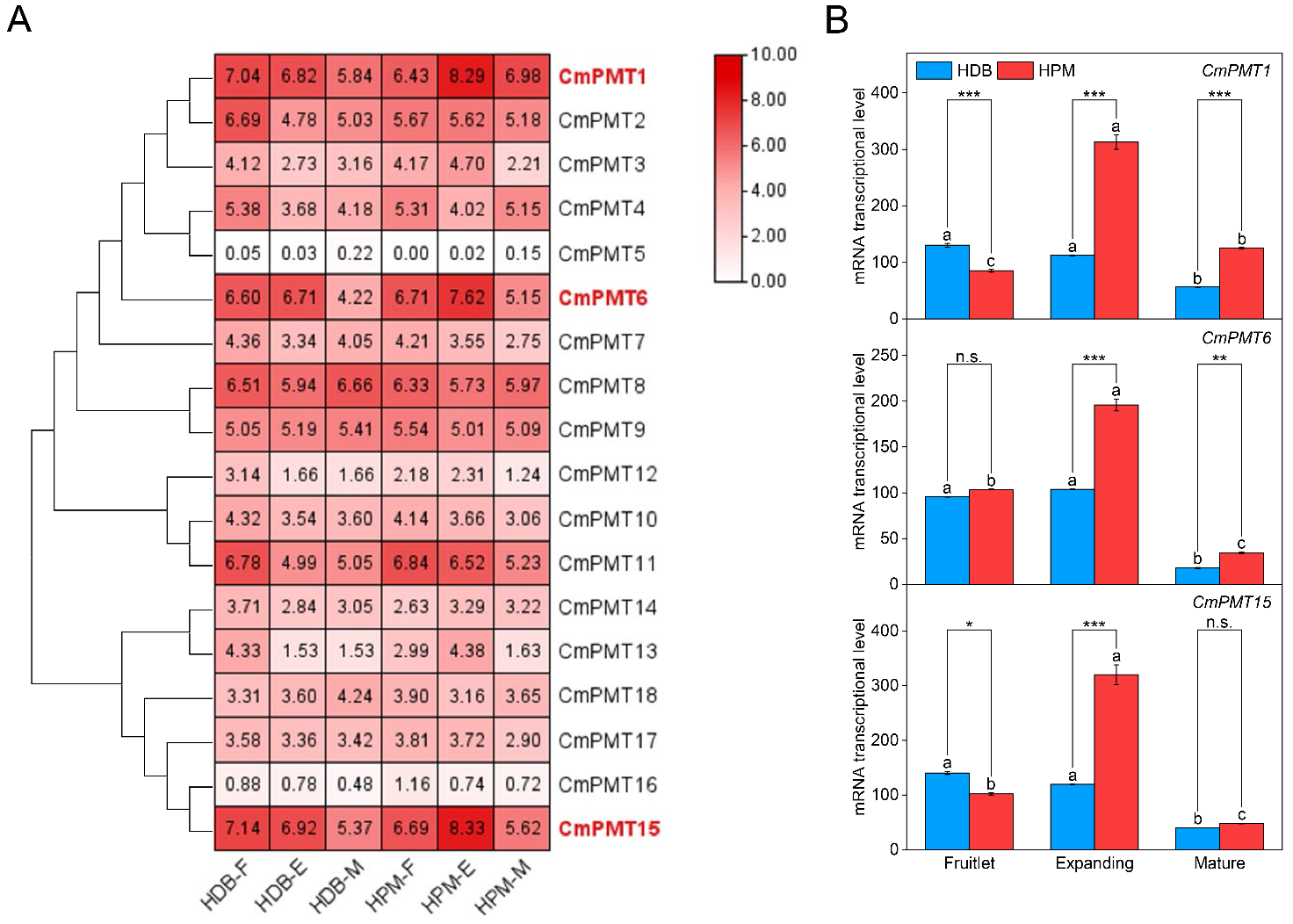


**Figure S8.** Gene expression pattern of *CmPMTs* during ‘HDB’ and ‘HPM’ fruit development. **A)** Heatmap of *CmPMTs* expression pattern. The gene expression of FPKM were log_2_-transformed. **B)** Differential analysis of *CmPMT1*, *CmPMT6* and *CmPMT15* expression in ‘HDB’ and ‘HPM’ fruit during development. Significant differences between the means were compared by Tukey test with * *P* < 0.05, ** *P* < 0.01 and *** *P* < 0.001. Different letters on each column indicate significant differences between the means among different developmental stages within each cultivar at *P* < 0.05 level.

**Figure S9.** Changes of cell wall material content in sarcocarp tissue after overexpression of *CmGoSAMT1*, *CmPMT1*, *CmPMT15* and *CmbZIP11* in ‘HPM’ fruit.


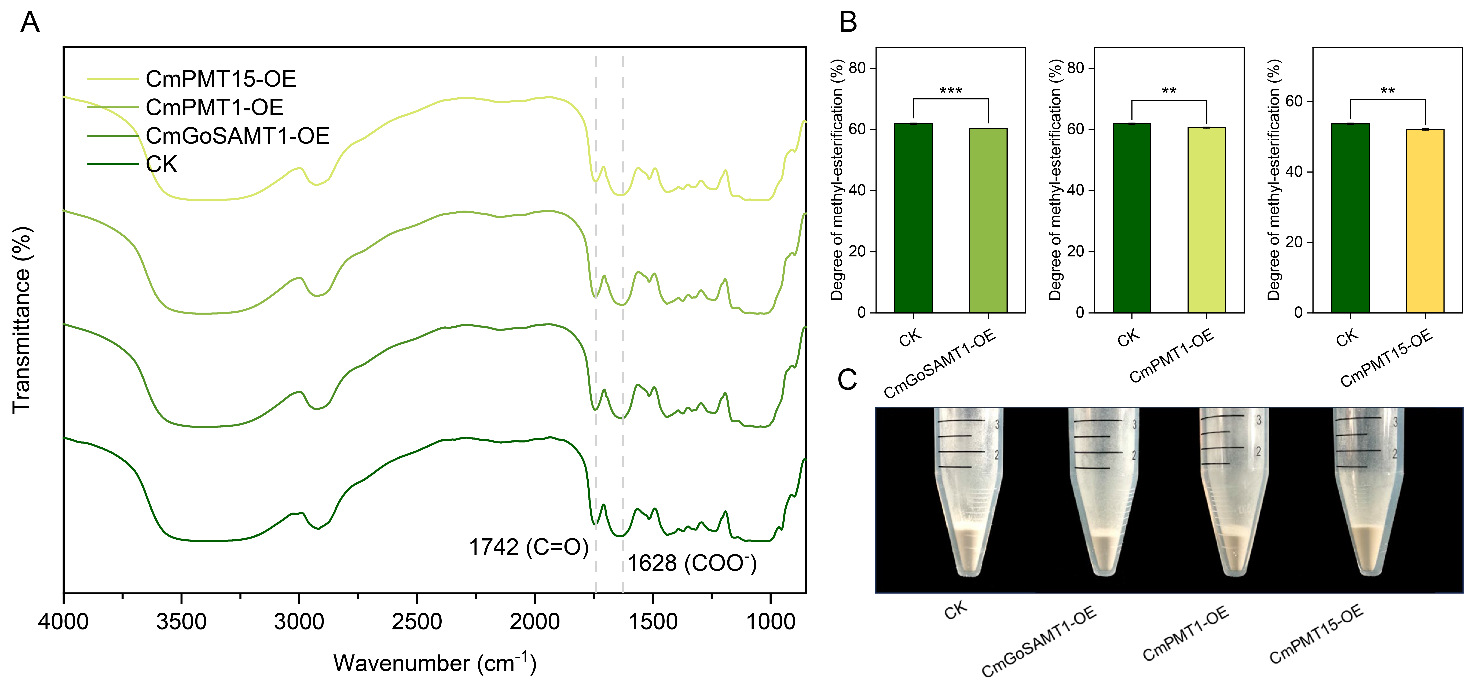


**Figure S10.** Effect on degree of methyl-esterification of HG after overexpression *CmGoSAMT1*, *CmPMT1* and *CmPMT15* in ‘HPM’ fruit. **A)** FT-IR spectra transmittance curves of CWM range from 4000 to 850 cm^-1^ after overexpression of *CmGoSAMT1*, *CmPMT1* and *CmPMT15* at 8.5 days after infection (DAI). The characteristic absorption peaks of C=O (1742 cm^-1^) and COO^-^ (1628 cm^-1^) on HG are annotated by gray dashed lines. **B)** Changes of HG degree of methyl-esterification after overexpression of *CmGoSAMT1*, *CmPMT1* and *CmPMT15* at 8.5 DAI. **C)** Cell wall material from sarcocarp tissue after overexpression of *CmGoSAMT1*, *CmPMT1* and *CmPMT15* at 8.5 DAI. Significant differences between the means were compared by Tukey test with * *P* < 0.05, ** *P* < 0.01 and *** *P* < 0.001.


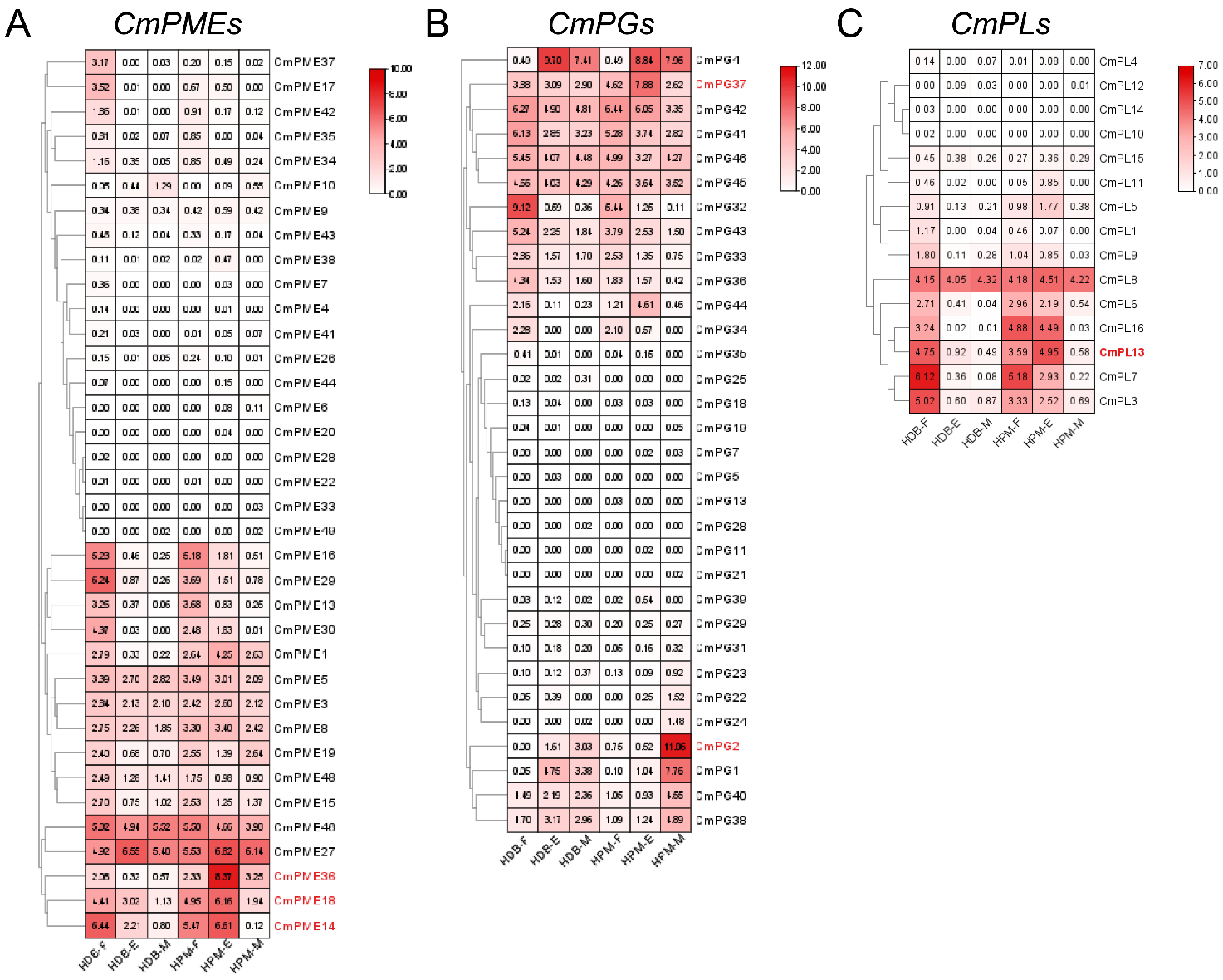


**Figure S11.** Gene expression pattern of *CmPMEs*, *CmPGs* and *CmPLs* during ‘HDB’ and ‘HPM’ fruit development. **A)** Heatmap of *CmPMEs* expression patterns. **B)** Heatmap of *CmPGs* expression patterns. **C)** Heatmap of *CmPLs* expression patterns. The gene expression of FPKM were log_2_-transformed.


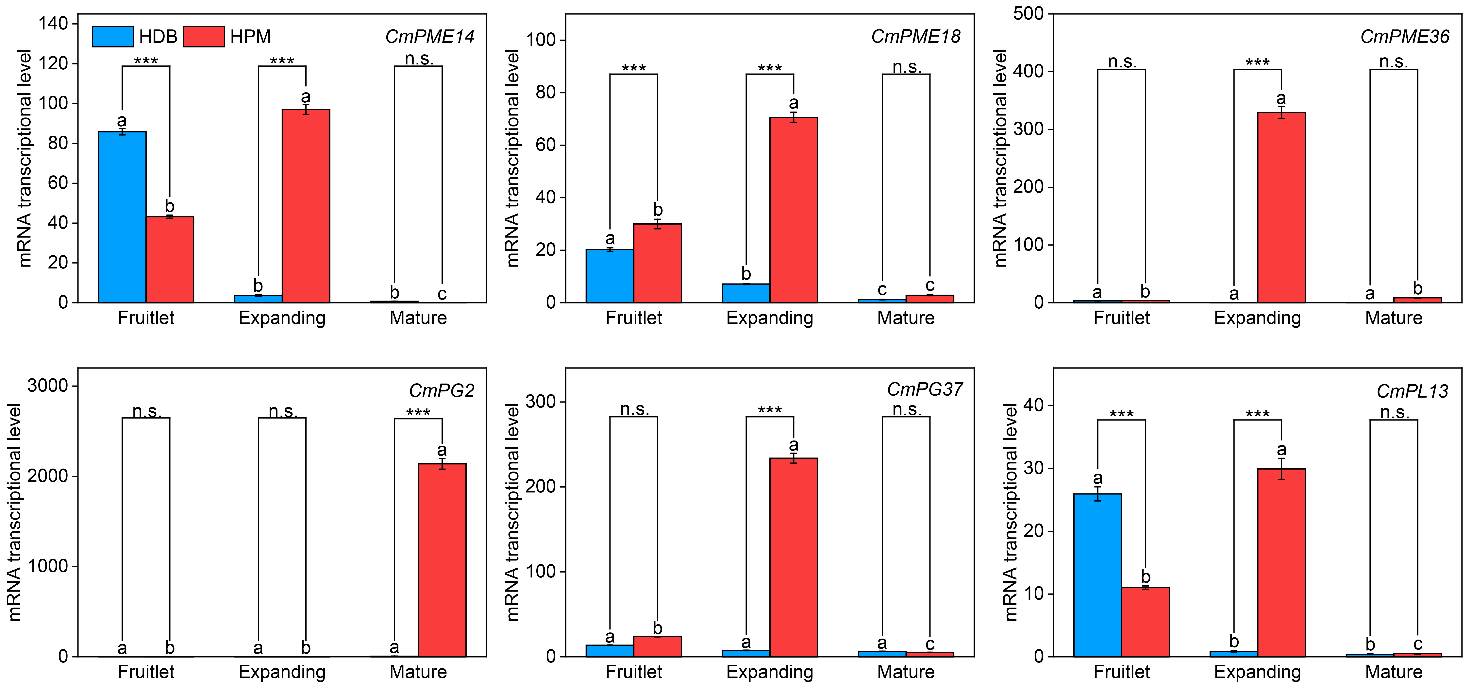


**Figure S12.** Differential analysis of *CmPMEs*, *CmPGs and CmPLs* members expression during ‘HDB’ and ‘HPM’ fruit development. Significant differences between the means were compared by Tukey test with * *P* < 0.05, ** *P* < 0.01 and *** *P* < 0.001. Different letters on each column indicate significant differences between the means among different developmental stages within each cultivar at *P* < 0.05 level.


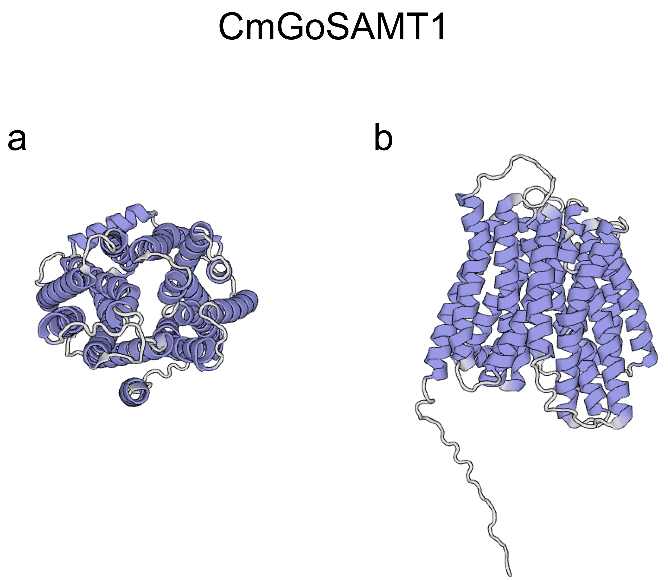


**Figure S13.** Protein three-dimensional structure of CmGoSAMT1.


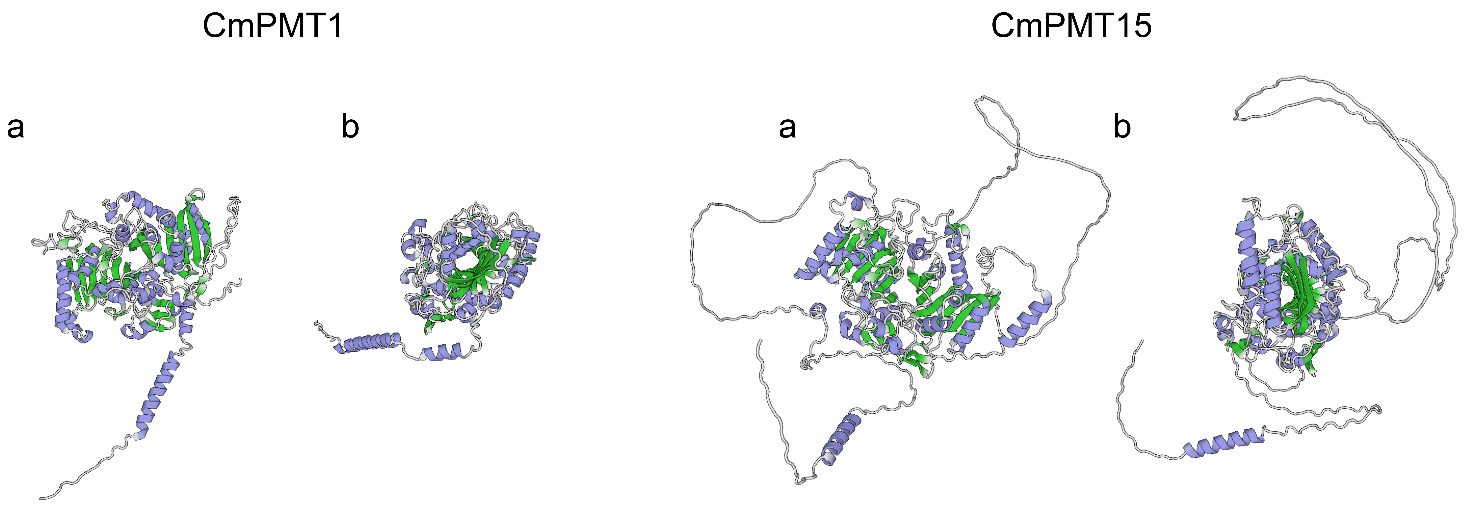


**Figure S14.** Protein three-dimensional structure of CmPMT1 and CmPMT15.


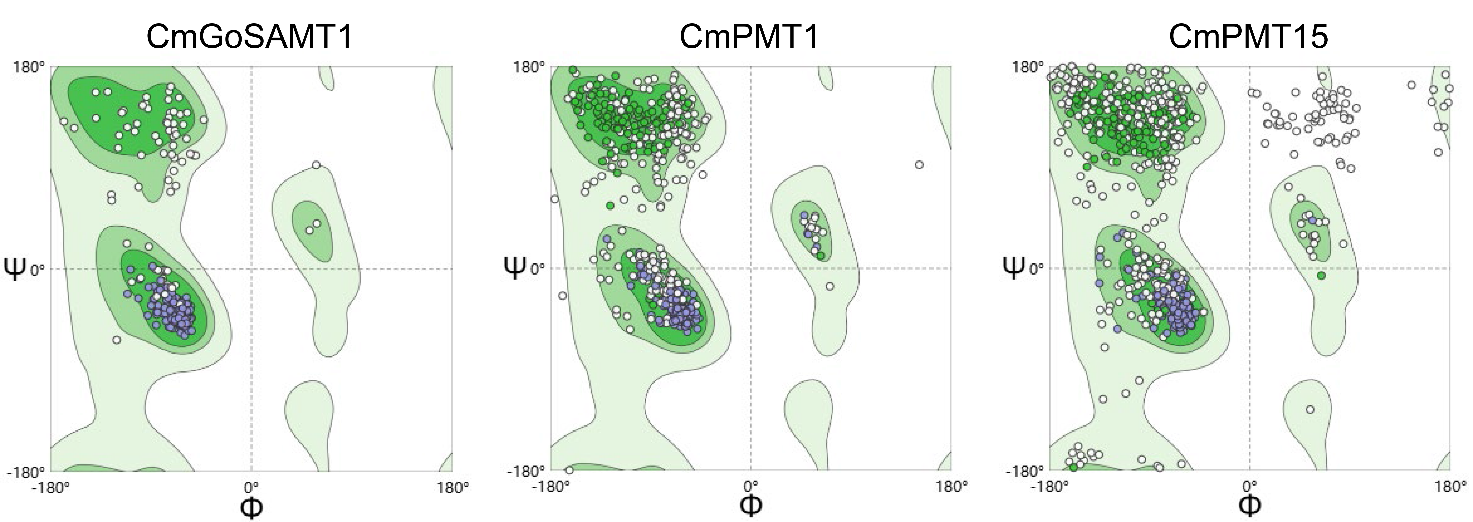


**Figure S15.** Ramachandran plots of CmGoSAMT1, CmPMT1 and CmPMT15 protein modeling structures.


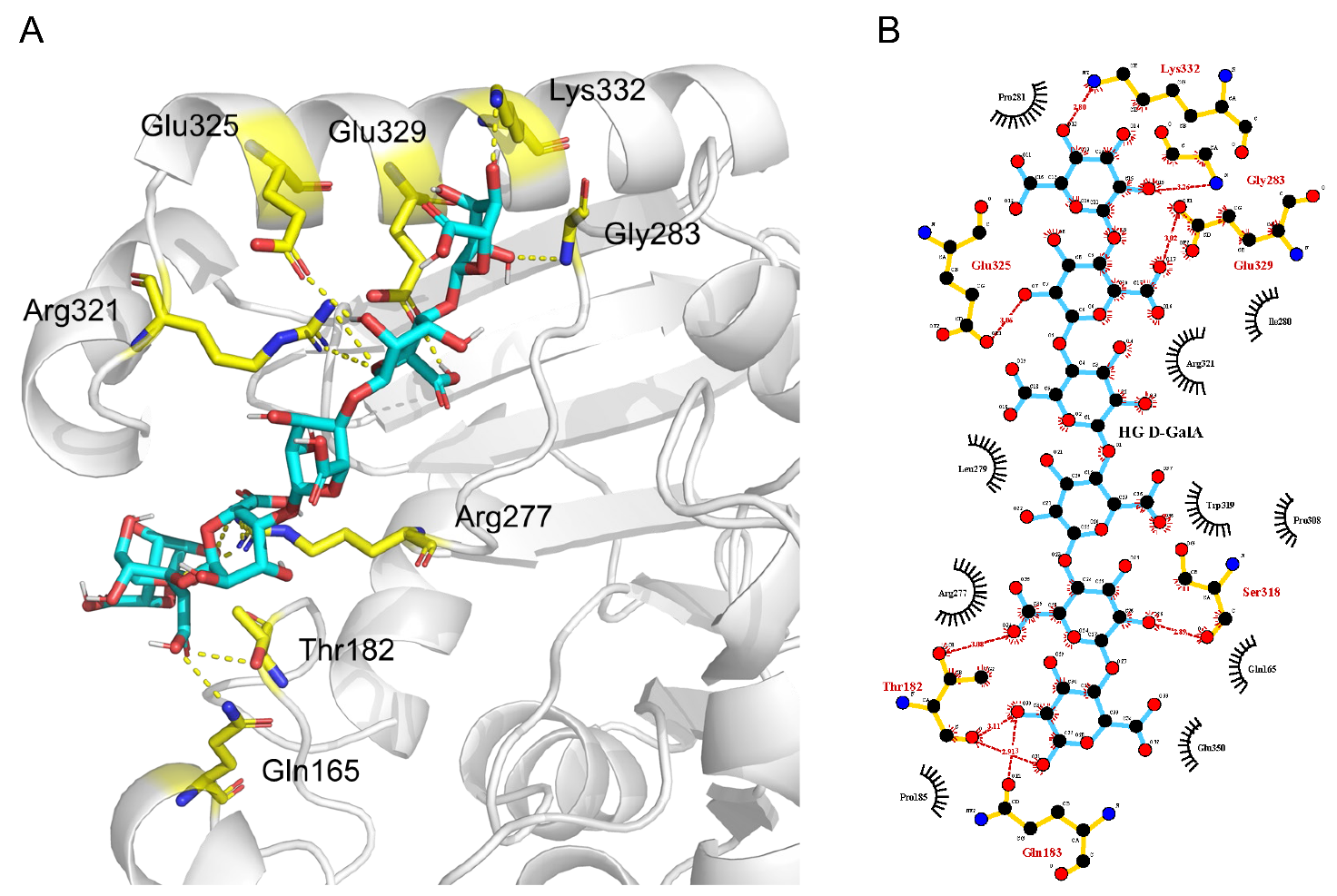


**Figure S16.** Molecular docking analysis of pectin methyltransferase (CmPMT1) with the ligand HG D-GalA. **A)** Spatial distribution of the amino acid residues interacting with a 6-unit 2_1_ helix D-GalA HG with hydrogen bonds in the active pocket. **B)** Schematic diagram representing the hydrogen bonding interaction between active amino acid residues with a 6-unit 2_1_ helix D-GalA HG.


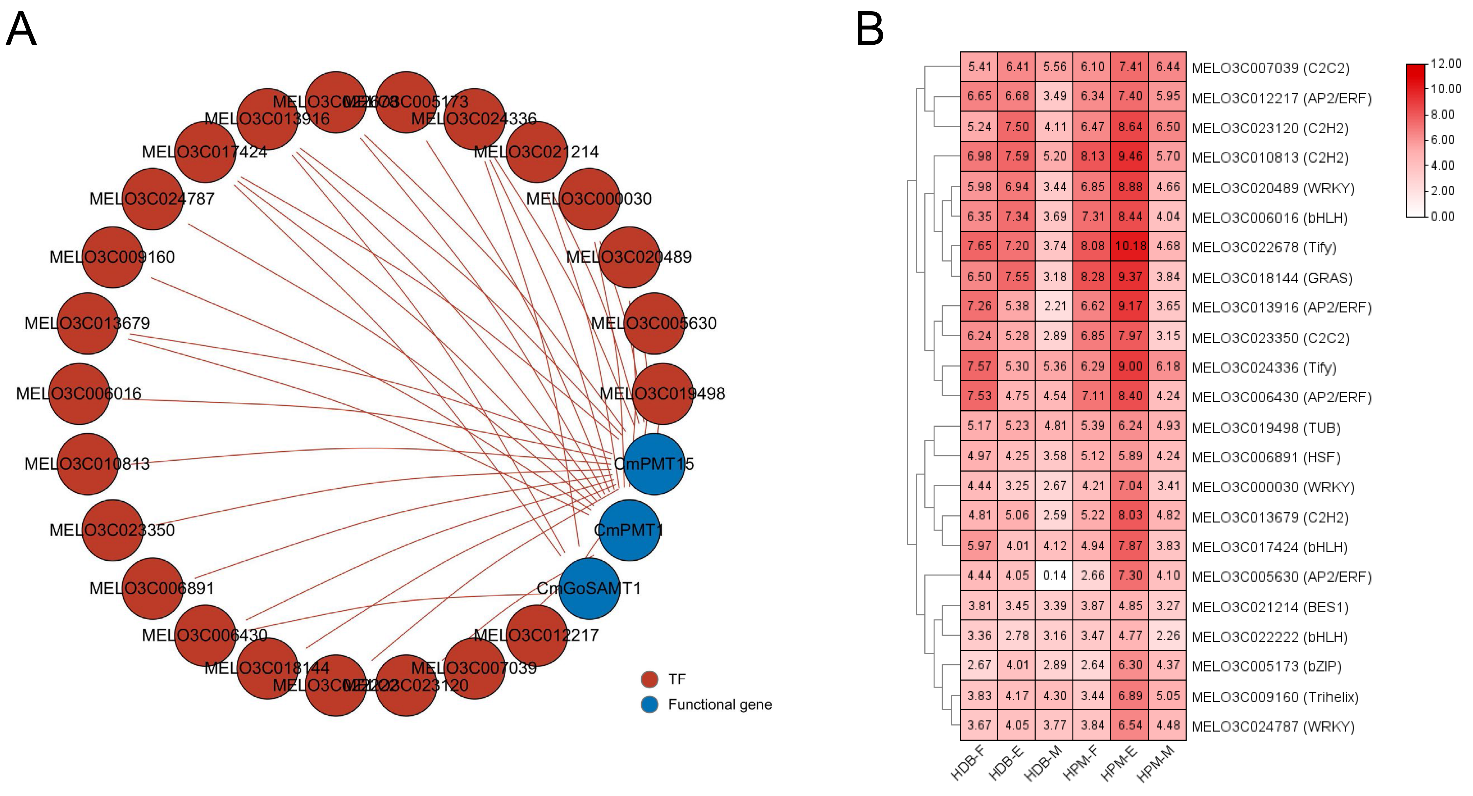


**Figure S17.** Expression pattern analysis of co-expressed transcription factors of *CmGoSAMT1*, *CmPMT1* and *CmPMT15.* **A)** Co-expression analysis between TFs with *CmGoSAMT1*, *CmPMT1* and *CmPMT15*. **B)** Expression pattern analysis of TFs. The gene expression of FPKM were log_2_-transformed.


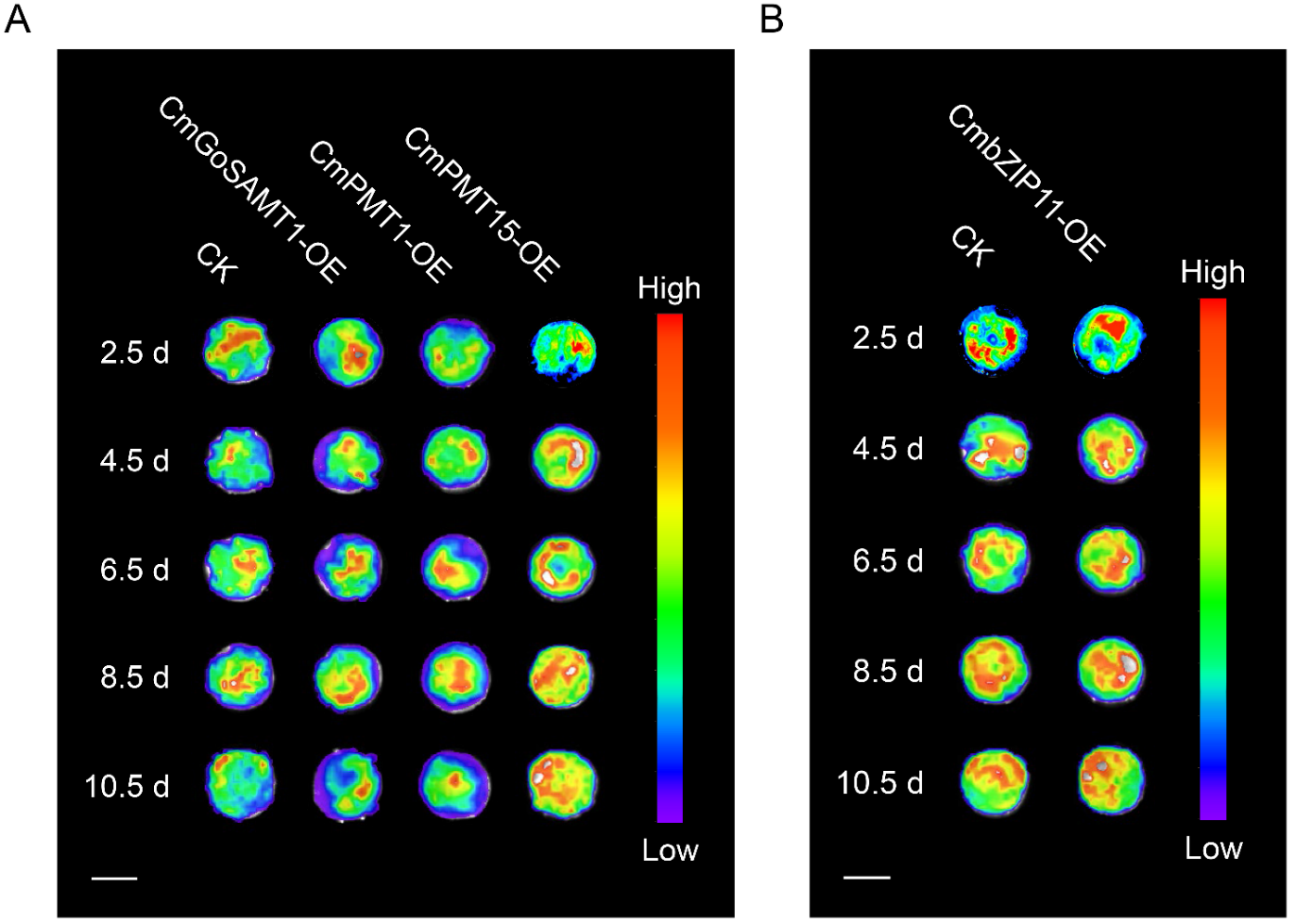


**Figure S18.** Fluorescence signal detection of sarcocarp tissue discs after overexpression of *CmGoSAMT1*, *CmPMT1*, *CmPMT15* and *CmbZIP11* in ‘HPM’ fruit. Scale bar = 1 cm.


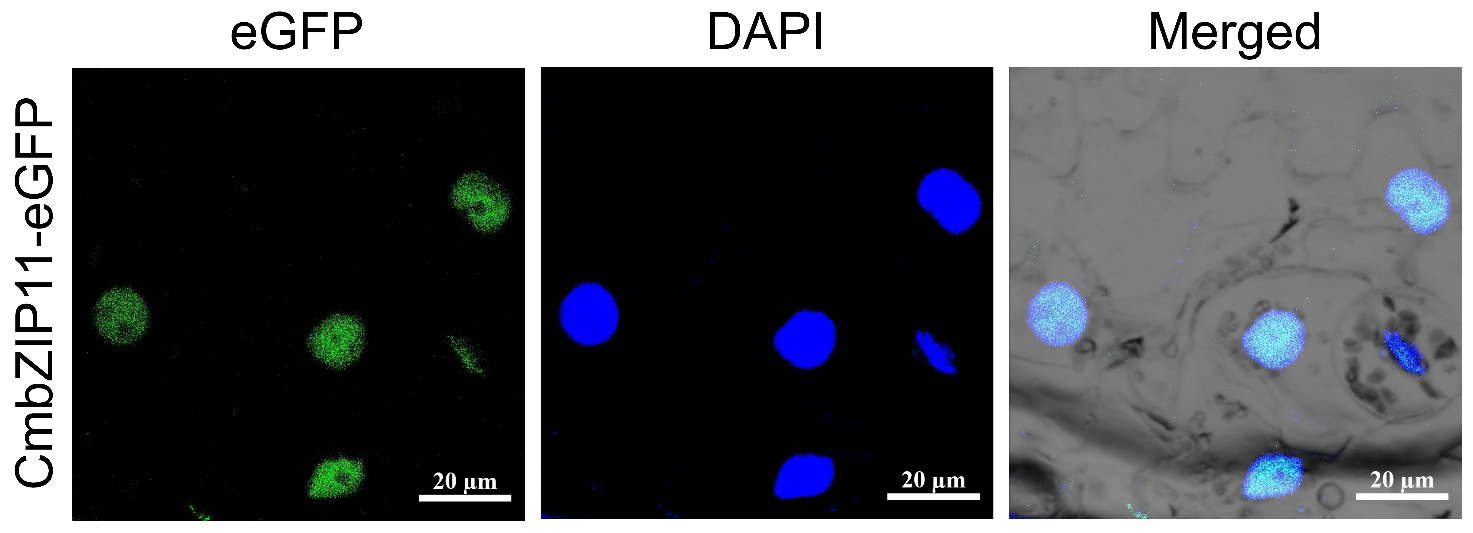


**Figure S19.** Subcellular localization of CmbZIP11 in the epidermis of tobacco leaves. Scale bar = 20 μm
