# Supplementary material for: CmbZIP11 regulates CmPMT1/15 affecting homogalacturonan methyl-esterification and fruit softening in melon
Source: Hortic Res. 2025 Sep 12;13(1):uhaf253. doi: 10.1093/hr/uhaf253 (PMC12881853; doi:10.1093/hr/uhaf253)
Supplement: Web_Material_uhaf253 [file web_material_uhaf253.zip › Supplementary Table.docx]

**T****able S1.** Gene ID of *CmGoSAMTs, CmPMTs*, *CmPMEs*, *CmPMEIs* and *CmSBTs* members in *Cucumis melo*

| Name | ID | Name | ID | Name | ID |
| --- | --- | --- | --- | --- | --- |
| *CmGoSAMT1* | MELO3C005898.1 | *CmPME40* | MELO3C014536.1 | *CmPMEI51* | MELO3C005291.1 |
| *CmGoSAMT2* | MELO3C007090.1 | *CmPME41* | MELO3C015550.1 | *CmPMEI52* | MELO3C017944.1 |
| *CmGoSAMT3* | MELO3C007612.1 | *CmPME42* | MELO3C023632.1 | *CmPMEI53* | MELO3C027867.1 |
| *CmPMT1* | MELO3C008535.1 | *CmPME43* | MELO3C024917.1 | *CmPMEI54* | MELO3C035537.1 |
| *CmPMT2* | MELO3C013875.1 | *CmPME44* | MELO3C010953.1 | *CmPMEI55* | MELO3C000551.1 |
| *CmPMT3* | MELO3C005813.1 | *CmPME45* | MELO3C023302.1 | *CmPMEI56* | MELO3C011973.1 |
| *CmPMT4* | MELO3C006144.1 | *CmPME46* | MELO3C022008.1 | *CmPMEI57* | MELO3C011974.1 |
| *CmPMT5* | MELO3C006882.1 | *CmPME47* | MELO3C012935.1 | *CmPMEI58* | MELO3C010682.1 |
| *CmPMT6* | MELO3C022753.1 | *CmPME48* | MELO3C004176.1 | *CmPMEI59* | MELO3C017910.1 |
| *CmPMT7* | MELO3C006141.1 | *CmPME49* | MELO3C020451.1 | *CmPMEI60* | MELO3C005567.1 |
| *CmPMT8* | MELO3C003569.1 | *CmPMEI1* | MELO3C030065.1 | *CmPMEI61* | MELO3C006821.1 |
| *CmPMT9* | MELO3C012145.1 | *CmPMEI2* | MELO3C027828.1 | *CmPMEI62* | MELO3C005566.1 |
| *CmPMT10* | MELO3C021595.1 | *CmPMEI3* | MELO3C027590.1 | *CmPMEI63* | MELO3C006820.1 |
| *CmPMT11* | MELO3C023001.1 | *CmPMEI4* | MELO3C025842.1 | *CmPMEI64* | MELO3C013975.1 |
| *CmPMT12* | MELO3C009779.1 | *CmPMEI5* | MELO3C019864.1 | *CmPMEI65* | MELO3C015549.1 |
| *CmPMT13* | MELO3C008236.1 | *CmPMEI6* | MELO3C001332.1 | *CmPMEI66* | MELO3C016001.1 |
| *CmPMT14* | MELO3C023888.1 | *CmPMEI7* | MELO3C027398.1 | *CmPMEI67* | MELO3C021070.1 |
| *CmPMT15* | MELO3C024603.1 | *CmPMEI8* | MELO3C019862.1 | *CmPMEI68* | MELO3C001969.1 |
| *CmPMT16* | MELO3C007878.1 | *CmPMEI9* | MELO3C030067.1 | *CmPMEI69* | MELO3C015291.1 |
| *CmPMT17* | MELO3C003837.1 | *CmPMEI10* | MELO3C031702.1 | *CmSBT1.10* | MELO3C005609.1 |
| *CmPMT18* | MELO3C018374.1 | *CmPMEI11* | MELO3C000643.1 | *CmSBT1.11* | MELO3C010808.1 |
| *CmPME1* | MELO3C015962.1 | *CmPMEI12* | MELO3C027585.1 | *CmSBT1.1a* | MELO3C018316.1 |
| *CmPME2* | MELO3C020953.1 | *CmPMEI13* | MELO3C019686.1 | *CmSBT1.1b* | MELO3C018317.1 |
| *CmPME3* | MELO3C003529.1 | *CmPMEI14* | MELO3C030072.1 | *CmSBT1.2* | MELO3C012837.1 |
| *CmPME4* | MELO3C012316.1 | *CmPMEI15* | MELO3C028017.1 | *CmSBT1.3* | MELO3C006851.1 |
| *CmPME5* | MELO3C006817.1 | *CmPMEI16* | MELO3C030077.1 | *CmSBT1.4* | MELO3C024346.1 |
| *CmPME6* | MELO3C005459.1 | *CmPMEI17* | MELO3C027265.1 | *CmSBT1.5* | MELO3C024314.1 |
| *CmPME7* | MELO3C016609.1 | *CmPMEI18* | MELO3C034829.1 | *CmSBT1.6* | MELO3C020810.1 |
| *CmPME8* | MELO3C006208.1 | *CmPMEI19* | MELO3C028007.1 | *CmSBT1.7a* | MELO3C007609.1 |
| *CmPME9* | MELO3C005292.1 | *CmPMEI20* | MELO3C028010.1 | *CmSBT1.7b* | MELO3C010817.1 |
| *CmPME10* | MELO3C006207.1 | *CmPMEI21* | MELO3C028113.1 | *CmSBT1.8* | MELO3C015526.1 |
| *CmPME11* | MELO3C021067.1 | *CmPMEI22* | MELO3C030063.1 | *CmSBT1.9* | MELO3C005605.1 |
| *CmPME12* | MELO3C021069.1 | *CmPMEI23* | MELO3C034865.1 | *CmSBT2.2* | MELO3C007040.1 |
| *CmPME13* | MELO3C022704.1 | *CmPMEI24* | MELO3C030064.1 | *CmSBT2.4* | MELO3C021725.1 |
| *CmPME14* | MELO3C024306.1 | *CmPMEI25* | MELO3C034823.1 | *CmSBT2.5* | MELO3C003980.1 |
| *CmPME15* | MELO3C007375.1 | *CmPMEI26* | MELO3C028122.1 | *CmSBT3.1* | MELO3C004753.1 |
| *CmPME16* | MELO3C005885.1 | *CmPMEI27* | MELO3C027589.1 | *CmSBT3.2* | MELO3C016358.1 |
| *CmPME17* | MELO3C015963.1 | *CmPMEI28* | MELO3C001272.1 | *CmSBT4.14a* | MELO3C018312.1 |
| *CmPME18* | MELO3C013699.1 | *CmPMEI29* | MELO3C027704.1 | *CmSBT4.14b* | MELO3C018314.1 |
| *CmPME19* | MELO3C015288.1 | *CmPMEI30* | MELO3C034867.1 | *CmSBT4.15a* | MELO3C024530.1 |
| *CmPME20* | MELO3C005196.1 | *CmPMEI31* | MELO3C032417.1 | *CmSBT4.15b* | MELO3C009456.1 |
| *CmPME21* | MELO3C005197.1 | *CmPMEI32* | MELO3C027703.1 | *CmSBT4.3* | MELO3C005832.1 |
| *CmPME22* | MELO3C005195.1 | *CmPMEI33* | MELO3C000722.1 | *CmSBT4.2* | MELO3C012343.1 |
| *CmPME23* | MELO3C006683.1 | *CmPMEI34* | MELO3C028053.1 | *CmSBT4.4* | MELO3C023899.1 |
| *CmPME24* | MELO3C021311.1 | *CmPMEI35* | MELO3C019860.1 | *CmSBT4.5* | MELO3C023903.1 |
| *CmPME25* | MELO3C015289.1 | *CmPMEI36* | MELO3C035196.1 | *CmSBT4.6* | MELO3C023912.1 |
| *CmPME26* | MELO3C022882.1 | *CmPMEI37* | MELO3C021270.1 | *CmSBT4.7* | MELO3C023917.1 |
| *CmPME27* | MELO3C022701.1 | *CmPMEI38* | MELO3C031526.1 | *CmSBT4.8* | MELO3C023918.1 |
| *CmPME28* | MELO3C002815.1 | *CmPMEI39* | MELO3C025616.1 | *CmSBT4.9* | MELO3C026563.1 |
| *CmPME29* | MELO3C023254.1 | *CmPMEI40* | MELO3C004166.1 | *CmSBT4.1* | MELO3C034026.1 |
| *CmPME30* | MELO3C023253.1 | *CmPMEI41* | MELO3C031308.1 | *CmSBT5.1* | MELO3C011392.1 |
| *CmPME31* | MELO3C027303.1 | *CmPMEI42* | MELO3C004168.1 | *CmSBT5.3* | MELO3C024176.1 |
| *CmPME32* | MELO3C002317.1 | *CmPMEI43* | MELO3C031307.1 | *CmSBT5.4a* | MELO3C026084.1 |
| *CmPME33* | MELO3C002915.1 | *CmPMEI44* | MELO3C031525.1 | *CmSBT5.4b* | MELO3C026085.1 |
| *CmPME34* | MELO3C016230.1 | *CmPMEI45* | MELO3C006266.1 | *CmSBT5.6* | MELO3C014071.1 |
| *CmPME35* | MELO3C009396.1 | *CmPMEI46* | MELO3C022458.1 | *CmSBT5.7* | MELO3C014075.1 |
| *CmPME36* | MELO3C005532.1 | *CmPMEI47* | MELO3C022457.1 | *CmSBT5.8* | MELO3C014076.1 |
| *CmPME37* | MELO3C015419.1 | *CmPMEI48* | MELO3C017187.1 | *CmSBT5.9* | MELO3C014082.1 |
| *CmPME38* | MELO3C026806.1 | *CmPMEI49* | MELO3C003367.1 | *CmSBT5.5* | MELO3C007179.1 |
| *CmPME39* | MELO3C031057.1 | *CmPMEI50* | MELO3C008049.1 | *CmSBT6* | MELO3C005470.1 |

**Table S2.** Gene ID of *AtGoSAMTs* and *AtPMTs* members in *Arabidopsis thaliana*

| ID | Name | ID | Name |
| --- | --- | --- | --- |
| AT1G64650.1 | *AtGoSAMT1* | AT5G04060.1 | **--** |
| AT4G27720.1 | *AtGoSAMT2* | AT3G10200.2 | **--** |
| AT3G49310.1 | *AtGoSAMT3* | AT5G06050.1 | **--** |
| AT2G45750.1 | *--* | AT2G39750.1 | **--** |
| AT4G00750.1 | *--* | AT1G77260.1 | **--** |
| AT1G33170.1 | *--* | AT5G14430.1 | **--** |
| AT4G10440.1 | *--* | AT4G14360.1 | **--** |
| AT4G18030.1 | *--* | AT3G23300.1 | **--** |
| AT1G26850.1 | *--* | AT1G04430.1 | **--** |
| AT4G19120.1 | *--* | AT1G19430.1 | **--** |
| AT1G31850.1 | *AtGMT1* | AT2G40280.1 | **--** |
| AT4G00740.1 | *AtGMT2/QUA3* | AT3G56080.1 | **--** |
| AT2G43200.1 | *--* | AT3G51070.1 | **--** |
| AT1G78240.1 | *AtQUA2/TSD2/OSU1* | AT5G64030.1 | **--** |
| AT1G13860.1 | *--* | AT2G34300.1 | **--** |
| AT2G03480.1 | *--* | AT1G29470.1 | **--** |

‘--’ represents unnamed.

**Table S3.** Primer for RT-qPCR

| Gene | Forward primer (5’-3’) | Reverse primer (5’-3’) |
| --- | --- | --- |
| *CmGoSAMT1* | CATCATGGACTGAGAACTATGG | CCTTCAAATAGAGACTGGATGG |
| *CmPMT1* | GGTACTATTAAGCTGCCTTACC | TTAGGTATGTTCCATCGTTTCC |
| *CmPMT15* | GAGAAGCCTGAAGAGAAACC | GTCCTCATTCTGTTCTTCGG |
| *CmbZIP11* | GGATAGTCGTCGATGAAAGG | CATCAAGATGCTTCTGTTTCC |
| *Cm18S* | AAACGGCTACCACATCCA | CACCAGACTTGCCCTCCA |

**Table S4.** Primer for gene cloning of CDS

| Gene | Forward primer (5’-3’) | Reverse primer (5’-3’) |
| --- | --- | --- |
| *CmGoSAMT1* | ATGGAGATTTTCTACTTCTTGG | CTAGATGTTTAACGGATCTGC |
| *CmPMT1* | ATGGCACCCAAACCAAGTTC | TCAGTGTGATGTGGAGTTGTTTC |
| *CmPMT15* | ATGGCATTGGGGAAGTATTC | CTAAGCAATTGCATATTGGAGT |
| *CmbZIP11* | ATGTTTTCTGCTTCCCTATC | CTACTCATCAGATTGGCTTG |

**Table S5.** Primer for various vectors

| Assey | Vector | Primer (5’-3’) |
| --- | --- | --- |
| Overexpression | CmGoSAMT1-3301LUC-F | GGACTCTTGACCATGGGATCC  ATGGAGATTTTCTACTTCTTGG |
|  | CmGoSAMT1-3301LUC-R | TTTGGCGTCTTCCATAAGCTT  GATGTTTAACGGATCTGCC |
|  | CmPMT1-3301LUC-F | GGACTCTTGACCATGGGATCC  ATGGCACCCAAACCAAGTTC |
|  | CmPMT1-3301LUC-R | TTTGGCGTCTTCCATAAGCTT  GTGTGATGTGGAGTTGTTTCCAC |
|  | CmPMT15-3301LUC-F | GGACTCTTGACCATGGGATCC  ATGGCATTGGGGAAGTATTC |
|  | CmPMT15-3301LUC-R | TTTGGCGTCTTCCATAAGCTT  AGCAATTGCATATTGGAGTGTT |
|  | CmbZIP11-3301LUC-F | GGACTCTTGACCATGGGATCC  ATGTTTTCTGCTTCCCTATC |
|  | CmbZIP11-3301LUC-R | TTTGGCGTCTTCCATAAGCTT  CTCATCAGATTGGCTTGAG |
| Y1H | CmPMT1pro-pABAi-F | GAAAAGCTTGAATTCGAGCTC  ATAGAGGAAGTGATCGTGGC |
|  | CmPMT1pro-pABAi-R | ATACAGAGCACATGCCTCGAG  TTGGAGTGGAGATCTCGG |
|  | CmbZIP11-pGADT7-F | GCCATGGAGGCCAGTGAATTC  ATGTTTTCTGCTTCCCTATC |
|  | CmbZIP11-pGADT7-R | CAGCTCGAGCTCGATGGATCC  CTACTCATCAGATTGGCTTG |
| Dual-LUC | CmPMT1pro-0800LUC-F | GTCGACGGTATCGATAAGCTT  AAAGTCTTTTCAAATTTGAGGATG |
|  | CmPMT1pro-0800LUC-R | CGCTCTAGAACTAGTGGATCC  GATGCGCCTGCATGGTTT |
|  | CmbZIP11-1300GFP-F | GAGCTCGGTACCCGGGGATCC  ATGTTTTCTGCTTCCCTATC |
|  | CmbZIP11-1300GFP-R | GCCCTTGCTCACCATGTCGAC  CTCATCAGATTGGCTTGAG |

**Table S6.** Correlation between *CmGoSAMT1*, *CmPMT1* and *CmPMT15* and their co-expressed transcription factors

| TFs | Functional gene | Correlation | *P*-value |
| --- | --- | --- | --- |
| MELO3C024336.1 (Tify) | *CmGoSAMT1* | 0.949 | 0.000 |
| MELO3C006430.1 (AP2/ERF) | *CmGoSAMT1* | 0.918 | 0.000 |
| MELO3C013916.1 (AP2/ERF) | *CmGoSAMT1* | 0.914 | 0.000 |
| MELO3C017424.1 (bHLH) | *CmGoSAMT1* | 0.912 | 0.000 |
| MELO3C005630.1 (AP2/ERF) | *CmPMT1* | 0.962 | 0.000 |
| MELO3C000030.1 (WRKY) | *CmPMT1* | 0.958 | 0.000 |
| MELO3C024336.1 (Tify) | *CmPMT1* | 0.954 | 0.000 |
| MELO3C005173.1 (bZIP) | *CmPMT1* | 0.953 | 0.000 |
| MELO3C017424.1 (bHLH) | *CmPMT1* | 0.949 | 0.000 |
| MELO3C024787.1 (WRKY) | *CmPMT1* | 0.946 | 0.000 |
| MELO3C009160.1 (Trihelix) | *CmPMT1* | 0.945 | 0.000 |
| MELO3C013679.1 (C2H2) | *CmPMT1* | 0.945 | 0.000 |
| MELO3C013916.1 (AP2/ERF) | *CmPMT1* | 0.933 | 0.000 |
| MELO3C020489.1 (WRKY) | *CmPMT1* | 0.929 | 0.000 |
| MELO3C019498.1 (TUB) | *CmPMT1* | 0.928 | 0.000 |
| MELO3C022678.1 (Tify) | *CmPMT1* | 0.928 | 0.000 |
| MELO3C021214.1 (BES1) | *CmPMT1* | 0.916 | 0.000 |
| MELO3C023120.1 (C2H2) | *CmPMT1* | 0.909 | 0.000 |
| MELO3C007039.1 (C2C2) | *CmPMT1* | 0.906 | 0.000 |
| MELO3C019498.1 (TUB) | *CmPMT15* | 0.963 | 0.000 |
| MELO3C020489.1 (WRKY) | *CmPMT15* | 0.958 | 0.000 |
| MELO3C021214.1 (BES1) | *CmPMT15* | 0.956 | 0.000 |
| MELO3C022678.1 (Tify) | *CmPMT15* | 0.953 | 0.000 |
| MELO3C013916.1 (AP2/ERF) | *CmPMT15* | 0.951 | 0.000 |
| MELO3C017424.1 (bHLH) | *CmPMT15* | 0.949 | 0.000 |
| MELO3C000030.1 (WRKY) | *CmPMT15* | 0.943 | 0.000 |
| MELO3C024336.1 (Tify) | *CmPMT15* | 0.941 | 0.000 |
| MELO3C006016.1 (bHLH) | *CmPMT15* | 0.936 | 0.000 |
| MELO3C010813.1 (C2H2) | *CmPMT15* | 0.930 | 0.000 |
| MELO3C023350.1 (C2C2) | *CmPMT15* | 0.921 | 0.000 |
| MELO3C006891.1 (HSF) | *CmPMT15* | 0.921 | 0.000 |
| MELO3C005630.1 (AP2/ERF) | *CmPMT15* | 0.918 | 0.000 |
| MELO3C013679.1 (C2H2) | *CmPMT15* | 0.917 | 0.000 |
| MELO3C018144.1 (GRAS) | *CmPMT15* | 0.917 | 0.000 |
| MELO3C006430.1 (AP2/ERF) | *CmPMT15* | 0.917 | 0.000 |
| MELO3C022222.1 (bHLH) | *CmPMT15* | 0.916 | 0.000 |
| MELO3C012217.1 (AP2/ERF) | *CmPMT15* | 0.904 | 0.000 |

**Table S7.** Active amino acid residue sites binding with SAM in PMT proteins

| Proteins | Active amino acid residue sites | | | | |
| --- | --- | --- | --- | --- | --- |
| AtQUA2/TSD2/OSU1 | Thr228 | Gly281 | Gly283 | Gln310 | Arg345 |
| AtQUA3 | Thr179 | Gly210 | Gly212 | Gln239 | Arg274 |
| CmPMT1 | Thr182 | Gly213 | Gly215 | Gln242 | Arg277 |
| CmPMT6 | Thr174 | Gly206 | Gly208 | Gln235 | Arg270 |
| CmPMT15 | Thr390 | Gly423 | Gly425 | Gln452 | Arg489 |
